# Topical 2′-Hydroxyflavanone for Cutaneous Melanoma

**DOI:** 10.3390/cancers11101556

**Published:** 2019-10-14

**Authors:** Chhanda Bose, Sharda P. Singh, Henry Igid, William C. Green, Sharad S. Singhal, Jihyun Lee, Philip T. Palade, Aditya Rajan, Somedeb Ball, Vijay Tonk, Ashly Hindle, Michelle Tarbox, Sanjay Awasthi

**Affiliations:** 1 Division of Hematology & Oncology, Department of Internal Medicine, Texas Tech University Health Sciences Center, Lubbock, TX 79430, USA; chhanda.bose@ttuhsc.edu (C.B.); henryigid@yahoo.com (H.I.); william.c.green@ttuhsc.edu (W.C.G.); jihyun.lee@ttuhsc.edu (J.L.); aditya.rajan@ttuhsc.edu (A.R.); somedeb.ball@ttuhsc.edu (S.B.); ashly.hindle@ttuhsc.edu (A.H.); 2 Department of Medical Oncology and Therapeutic Research, City of Hope National Medical Center, Duarte, CA 91010, USA; ssinghal@coh.org; 3 Department of Pharmacology and Toxicology, University of Arkansas for Medical Sciences, Little Rock, AR 72205, USA; ppalade@uams.edu; 4 Department of Pediatrics, Texas Tech University Health Sciences Center, Lubbock, TX 79430, USA; Vijay.tonk@ttuhsc.edu; 5 Department of Dermatology and Dermatopathology, Texas Tech University Health Sciences Center, Lubbock, TX 79430, USA; michelle.tarbox@ttuhsc.edu

**Keywords:** 2′-hydroxyflavanone, melanoma, pro-apoptotic signaling, topical application, Ralbp1, RLIP76

## Abstract

2′-hydroxyflavanone (2HF) is a dietary flavonoid with anticancer activity towards multiple cancers. Here, we report that topically applied 2HF inhibits the growth of intradermal implants of melanoma in immunocompetent mice. 2HF induced apoptosis and inhibited the growth of the human SK-MEL-24 as well as murine B16-F0 and B16-F10 melanoma cell lines in vitro. Apoptosis was associated with depletion of caspase-3, caspase-9, and PARP1 in B16-F0 and SK-MEL-24 cells. Caspase-9 and MEKK-15 were undetected even in untreated B16-F10 cells. Signaling proteins TNFα, and phospho-PDGFR-β were depleted in all three cell lines; MEKK-15 was depleted by 2HF in SK-MEL-24 cells. 2HF enhanced sunitinib (an MEK and PDGFR-β inhibitor) and AZD 2461 (a PARP1 inhibitor) cytotoxicity. 2HF also depleted the Ral-regulated, stress-responsive, antiapoptotic endocytic protein RLIP76 (RALBP1), the inhibition of which has previously been shown to inhibit B16-F0 melanoma growth in vivo. Functional inhibition of RLIP76 was evident from inhibition of epidermal growth factor (EGF) endocytosis by 2HF. We found that topically applied 2HF–Pluronic Lecithin Organogel (PLO) gel inhibited B16-F0 and B16-F10 tumors implanted in mice and caused no overt toxicity despite significant systemic absorption. 2HF treatment reduced phospho-AKT, vimentin, fibronectin, CDK4, cyclinB1, and BCL2, whereas it increased BIM and phospho-AMPK in excised tumors. Several cancer signals are controlled by endocytosis, a process strongly inhibited by RLIP76 depletion. We conclude that 2HF–PLO gel may be useful for topical therapy of cutaneous metastases of melanoma and could enhance the antineoplastic effects of sunitinib and PARP1 inhibitors. The mechanism of action of 2HF in melanoma overlaps with RLI76 inhibitors.

## 1. Introduction

Though most cancers can metastasize to the skin [1,2,3], melanoma has a particularly strong predilection for giving rise to regional or distant skin metastases. Before effective immunotherapies, autopsy studies found skin metastases in a striking 75% of patients dying of melanoma [4]. Despite earlier diagnosis and effective immunotherapy, the incidence of skin metastases continues to be quite high even in the present day, with perhaps one-fifth to half of late stage patients having skin metastases [5]. Because skin, lymphatic, and nodal metastases are most often reported as an aggregate [6,7], the true frequency of skin metastases is likely lower, but still comparable to that in breast cancer where up to a fourth of patients with advanced metastatic cancer have developed skin metastases prior to death [1,2,3,5,7,8]. Surgical resection and/or radiation constitute the mainstay of palliative therapy for skin metastases, but physicians treating advanced melanoma are frequently confronted with recurrent, large, aggressively growing, painful, ulcerated, and treatment-resistant skin metastases for which satisfactory palliative therapies are lacking. Local therapies such as cryoablation, topical imiquimod or 5-fluorouracil, intralesional injections of methotrexate, interleukin-2, interferon, or BCG (Bacillus Calmette–Guerin) often have sub-optimal activity and unacceptable local toxicity. The oncolytic virus *talimogene laherparepvec* can be quite effective, but the majority of patients fail to respond and its use can be limited by the side effects of cytokine release syndrome [9]. Patients with extensive involvement of the extremities can benefit from isolated limb perfusion with L-phenylalanine mustard, but this approach is costly and is associated with significant systemic toxicity [10]. Systemic immunotherapies with checkpoint inhibitors or high-dose IL2 are active against skin metastases, but also limited by toxicity and primary or acquired resistance. Thus, more effective and less toxic options are necessary for palliation of the morbidity caused by cutaneous metastases of melanoma.

Numerous phytochemicals from edible sources have been shown to have antineoplastic properties and are ‘generally regarded as safe′ (GRAS) by the Food and Drug Administration [11,12]. We have shown that 2-hydroxyflavanone (2HF), a nontoxic flavonoid phytochemical abundant in orange peel, exerts antineoplastic effects on breast, renal, and lung carcinoma [13,14,15,16]. It impairs the mercapturic acid pathway (MaP), which is responsible for defending cancer cells against apoptosis caused by exogenous toxins such as chemotherapy drugs (xenobiotics) and endogenous pro-apoptotic metabolites (endobiotics) such as 4-hydroxynonenal (4HNE) derived from oxidation of ω-6 fatty acids.

2HF reduces the activity and expression of two key enzymes of MaP, glutathione S-transferase (GST) and RLIP76 (also known as RALBP1 or Rlip) [16]. GST catalyzes the conjugation of glutathione (GSH) to electrophilic (alkylating) xenobiotics and endobiotics. The resultant glutathione–electrophile conjugates (GS-E) are removed from cells through ATP-dependent efflux from cells by Rlip, a stress-protective membrane ATPase that couples ATP-dependent efflux of GS-E to clathrin-dependent endocytosis (CDE) of ligand–receptor complexes in plasma membranes [17]. The central role of Rlip in providing protection from apoptotic stress is evident from studies of Rlip-null mice that are highly sensitive to stress caused by toxic electrophiles as well as radiation, both of which can cause apoptosis through generation of 4HNE [18,19]. This strong anti-apoptotic function is also important for the survival of cancer cells, as evident from multiple in vivo studies showing that inhibition of Rlip by antibodies or depletion by antisense causes regression of melanoma, neuroblastoma, and carcinomas of the lung, kidney, prostate, pancreas, and colon in immunocompetent and nude mouse models [13,17,20,21,22,23,24,25,26,27]. Rlip null mice also have impaired CDE; thus ligand–receptor signaling mechanisms important for carcinogenesis, diabetes, and obesity are also deficient in these mice, explaining their remarkable resistance to chemical carcinogenesis [17], diabetes, metabolic syndrome [28], and obesity [29]. We have recently shown that Rlip haploinsufficiency switches off the spontaneous carcinogenesis phenotype of p53-null mice, which normally develop malignancy in 100% of animals before the age of six months; no prior pharmacological or genetic intervention had previously achieved this [30]. These paradigm-shifting studies, showing that spontaneous carcinogenesis upon loss of the tumor suppressor functions of p53 can be bypassed by Rlip deficiency, have established an existential importance of stress resistance and CDE functions of Rlip for cancer cell survival.

Because 2HF inhibits Rlip, and targeted inhibition of Rlip regresses melanoma in vivo [31], it followed logically that 2HF would exert anti-melanoma activity. Because of the relatively poor oral pharmacological properties of 2HF, and the need for palliative topical therapies for melanoma skin metastases, we addressed our hypothesis by testing a topical formulation of 2HF in an immunocompetent model of mouse melanoma. Our results show that topical application of 2HF–Pluronic Lecithin Organogel (PLO) gel inhibits cutaneous implants of melanoma by inhibiting multiple melanoma growth pathways. Because Rlip catalyzes sunitinib efflux and its downstream targets overlap with Rlip inhibitors [23], and because of recent transcriptomic evidence that PARP1 expression is suppressed by Rlip deficiency, we performed in vitro studies of the combined effects of 2HF with sunitinib and the PARP1 inhibitor AZD 2461. Our findings provide a strong rationale for the development of 2HF as a topical therapy and lay the groundwork for the combination with sunitinib or PARP1 inhibitors in the treatment of melanoma.

## 2. Results

### 2.1. 2HF Inhibited the Growth/Survival of Melanoma Cell Lines in Vitro

**Figure 1:**
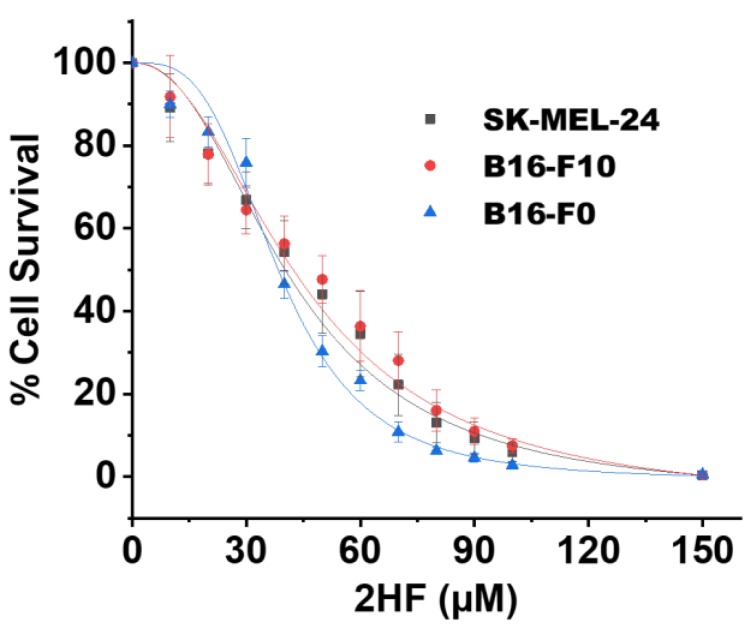
Mouse and human melanoma cell survival following 2′-hydroxyflavanone (2HF) treatment. Drug sensitivity assays were performed by 3-(4,5-dimethylthiazol-2-yl)-2,5-diphenyltetrazolium bromide (MTT) assay at 72 h post treatment with 2HF to determine IC_50_. Values are presented as mean ±SD from five separate determinations with eight replicates each (*n* = 5).

We studied the growth-inhibitory effects of 2HF on the B16-F0 and B16-F10 murine melanoma cell lines as well as on the SK-MEL-24 human melanoma cell line. B16-F10 and SK-MEL-24 have been shown in previous studies to be highly resistant to treatment and much more invasive than B16-F0 [32]. We found that 2HF inhibited the growth/survival of B16-F0, B16-F10, and SK-MEL-24 cells nearly equally (IC_50_ at 72 h: B16-F0: 38 ± 4 µM, B16-F10: 47 ± 6 µM, and SK-MEL-24: 44 ± 6 µM) using the spectrophotometric 3-(4,5-dimethylthiazol-2-yl)-2,5-diphenyltetrazolium bromide (MTT) assay (Figure 1). The absorption value measured upon reduction of the MTT dye by functional mitochondria is an indirect measure of cell number that cannot distinguish inhibited cell proliferation from apoptotic or necrotic cell death. By counting viable (trypan-blue dye-excluding) cells using a hemacytometer, we verified that 2HF did indeed cause cell death.

### 2.2. 2HF-Induced Apoptosis in Melanoma Cell Lines in Vitro

**Figure 2:**
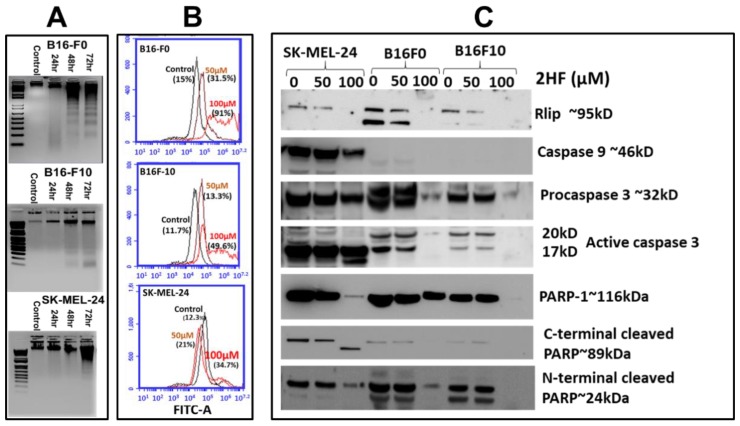
(**A**) 2HF-induced apoptosis detected by DNA laddering in the B16-F0, B16-F10, and SK-MEL-24 cell lines. Mouse and human melanoma cells were incubated with or without 50 µM 2HF for 24–72 h, washed, and harvested. DNA was isolated and processed as described in Materials and Methods Section. Lane 1: Standard molecular size marker (1 Kb). Lane 2: Untreated cells, Lanes 3–5: 2HF- treated cells. (**B**) Effect of 2HF on DNA fragmentation in B16-F0, B16-F10, and SK-MEL- 24 cells as measured by terminal deoxynucleotidyl transferase dUTP-mediated nick-end labeling (TUNEL) assay. Cells were treated with different doses of 2HF for 48 h, as described in Materials and Methods. After treatments, apoptotic intensity of cells was determined by flow cytometry. Histograms show the number (counts) of TUNEL-positive cells in different groups. Data are shown as a logarithmic histogram and expressed as fluorescence intensity of number of counts of the TUNEL-positive cells obtained from the statistical analysis of the fluorescence height and mean value of the x-axis. (**C**) Effect of 2HF on RLIP and the activation of caspases. Melanoma cells were treated with 50 or 100 µM 2HF for 48 h, and then whole-cell lysates were prepared and subjected to Western blotting using antibodies against the indicated proteins.

The DNA laddering assay was used to examine whether cell death caused by 2HF was accompanied by apoptosis. In this assay, apoptosis is evident in agarose gels that show a DNA ladder in which each rung is 180 bp shorter than the previous, the consequence of sequential DNA cleavage by caspase-activated DNAase during the execution phase of apoptosis [33]. 2HF-induced apoptosis was confirmed by the appearance of DNA ladders in SYBR-Safe (Invitrogen)-stained agarose gels of nuclear extracts of B16-F0, B16-F10, and SK-MEL-24 cells treated with 50 μM 2HF. The intensity of staining increased in a time-dependent manner at 24, 48, and 72 h (Figure 2A). Appearing first as a smear at 24 h, DNA laddering was distinctly evident by 48 and 72 h after treatment. Apoptosis was confirmed by fluorescence-activated cell sorting (FACS) analysis using the APO-BrdU™ terminal deoxynucleotidyl transferase dUTP-mediated nick-end labeling (TUNEL) Assay Kit, which detects terminal deoxynucleotidyl transferase (TdT)-mediated nick-end labeling. Quantitative analysis of the counts of TUNEL-positive cells of each treatment demonstrated that B16-F0, B16-F10, and SK-MEL-24 cells were sensitive towards 2HF treatment (Figure 2B). Apoptosis appears to have contributed more clearly to the 2HF-induced decrease in survival more in B16-F0 cells than in B16-F10 or SK-MEL-24 cells, which showed little increase in DNA laddering (B16-F10) or TUNEL positivity (SK-MEL-24), indicating that other cell death mechanisms in addition to apoptosis may be operative following 2HF treatment in melanoma.

### 2.3. 2HF Depletes Rlip and PARP1 and Influences the Cytotoxic Effects of PARP1 Inhibitor AZD 2461

To determine the effects of 2HF on apoptosis we performed western blots on caspases and PARP1, and to assess possible contributions of the mercapturic acid pathway, we assessed expression of Rlip. As expected, depletion of Rlip by 2HF treatment was confirmed in all three cell lines (Figure 2C). Caspase-9 was clearly expressed in SK-MEL-24 but was undetectable in B16-F10 and only barely detectable in B16-F0. Procaspase-3 and activated caspase-3 were detectable in all three cell lines. An increase in cleaved caspase-3 with corresponding depletion of procaspase-3 can be seen in the human cell line SK-MEL-24, most evidently at 100 µM 2HF, while for the two murine cell lines, the overall expression of caspase-3 was diminished. Interestingly PARP1 expression was present in all three cell lines and the overall expression level was greatly reduced by 2HF in the models with greater resistance. At 100 µM 2HF, PARP1 disappeared in B16-F10, was greatly reduced in SK-MEL-24, but was less affected in B16-F0. These results were also reflected in the C- and N-terminal cleavage fragments of PARP1; however, the overall depletion of PARP1 rendered the fragments uninformative as an apoptotic marker (Figure 2C). Taken together, these results also suggest the involvement of apoptosis in conjunction with other novel mechanisms. We also cannot discount possible differences in mouse and human cell physiology, and further studies will need to be done in additional human models to address this.

Though PARP1 cleavage has been used primarily as a marker of apoptosis, the recent use of PARP1 inhibitors for therapy of homologous recombination-dependent DNA repair-deficient (constitutionally Breast Cancer gene [BRCA]1/2-deficient) cancers has shed new light on the mechanistic significance of differences in the sensitivity to PARP1-cleavage between cancer cells of differing sensitivity to therapy. Specifically, cells with lower PARP1 expression could be differentially sensitive to PARP1 inhibitors. In light of the potential for PARP1 inhibitors as therapeutics in melanoma [34,35,36,37,38,39,40], and the striking depletion of cellular PARP1 observed following 2HF treatment, we considered the possibility that 2HF could modulate the sensitivity of melanoma to PARP1 inhibitors. Thus, we investigated the cytotoxicity of PARP1 inhibitor AZD 2461 in these cell lines with or without 20 µM 2HF. SK-MEL-24 is known to be BRCA2 wild-type while B16-F10 is BRCA2 deficient (K1997N mutation). Our results suggested the potentiation of the PARP1 inhibitor by 2HF was numerically greater in both murine cell lines than SK-MEL-24 (Table 1). Since the PARP1 level was qualitatively higher in SK-MEL-24 than either murine cell line, the results suggested that 2HF could potentiate PARP1 inhibitor efficacy preferentially in cell lines having lower levels of PARP1, thus lower levels of non-homologous end joining (NHEJ) DNA repair. Low levels of BRCA1 and BRCA2 in all cell lines did not allow any conclusions regarding the role of this DNA-repair protein in the differential effects of 2HF on PARP1 inhibitor cytotoxicity. The validity and potential clinical relevance of this observation needs to be explored further in future studies.

### 2.4. 2HF Inhibits Endocytosis as Effectively as Depletion of Rlip

To determine whether the 2HF-induced reduction of Rlip protein had functional implications, we examined whether 2HF inhibited CDE in melanoma in a manner similar that we had observed recently in lung cancer [14]. Rlip is an important component of clathrin-dependent endocytosis (CDE) [17]. It is bound to the clathrin coat through the AP2 adaptor protein and is thought to provide energy for CDE through ATP hydrolysis that is coupled to efflux of GS-X [17]. Formation of the Rlip/Clathrin/AP2/POB1 signalosome allows vesicle formation and subsequent endocytosis of activated epidermal growth factor (EGF) receptors [41,42]. Mouse embryonic fibroblasts (MEFs) from Rlip-null mice are deficient in CDE [30]. We have also shown that the rate of GS-X efflux by Rlip mutants correlates directly with its anti-apoptotic activity and the rate of endocytosis of EGF and insulin [17]. 2HF causes regression of renal cell carcinoma xenografts with signaling effects similar to those seen upon treatment with Rlip-specific antisense, siRNA, or anti-Rlip antibodies [23,43]. Furthermore, we have recently reported that 2HF binds directly to Rlip, inhibiting its transport activity and potentiating the anticancer activity of anti-Rlip antibodies in lung cancer, indicating that Rlip inhibition is a mechanism of action of 2HF [14].

**Figure 3:**
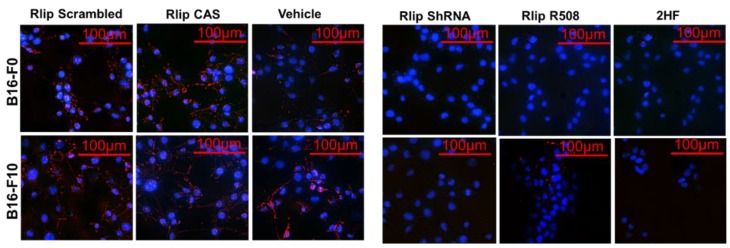
Effects of Rlip depletion on epidermal growth factor (EGF) endocytosis of mouse melanoma cells. Representative photomicrographs of Rlip-depleted B16-F0 and B16-F10 cells showing the inhibition of EGF-pHrodo endocytosis. EGF-pHrodo endocytosis is completely blocked in Rlip-depleted B16-F0 and B16-F10 cells. Rlip was depleted by treating cells with Rlip-antisense, Rlip-shRNA, or 10 µM 2HF for 24 h as described in Materials and Methods section 4.8. Photographs were taken at identical exposures using 200× magnification.

**Figure 4:**
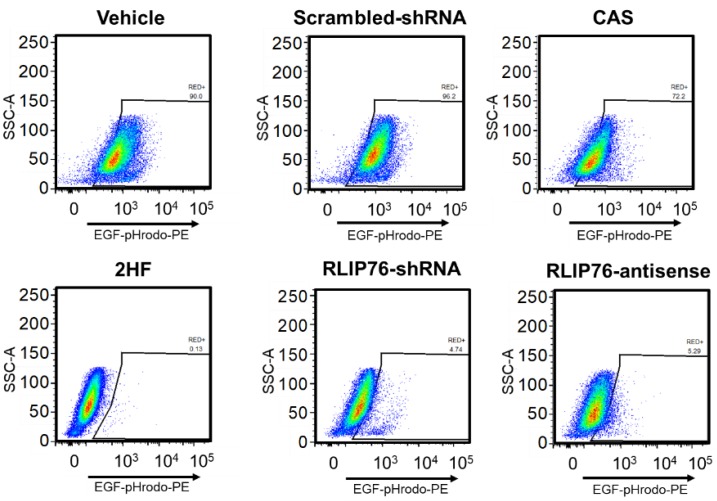
Flow cytometry analysis of EGF-pHrodo endocytosis in control or Rlip depleted B16-F0 cells. Flow cytometry analysis of B16-F0 cells was performed 24 h after Rlip depletion by shRNA, RLIP76-antisense, or treatment with 100 µM 2HF. Cells were stained with EGF-pHrodo. EGF-pHrodo-positive cells as shown on scatter plots were identified and gated based on forward and side scatter properties. The PE channel was used for red fluorescence to measure EGF-pHrodo positivity. Data are shown as logarithmic dot plots and expressed as mean fluorescence intensity, as obtained from the statistical analysis of the fluorescence height and mean value of the x-axis as displayed by the software. Each flow cytometry analysis plot is a representative example of three independent experiments.

**Figure A1:**
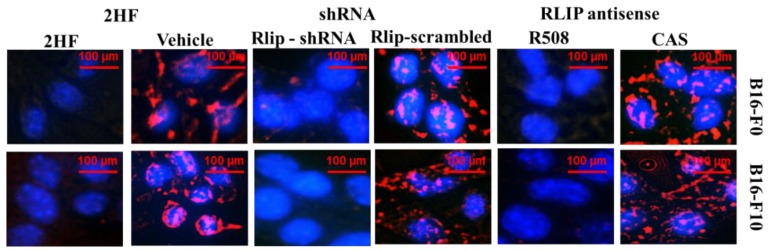
Effects of Rlip depletion on EGF endocytosis of B16-F0 and B16-F10 cells. High magnification views are shown demonstrating the inhibitory effect of 100µM 2HF and of Rlip-depletion on endocytosis of EGF-pHrodo. Procedures used are explained in Materials and Methods Section.

To determine whether 2HF-mediated blockade of Rlip function was also relevant in melanoma, we tested whether Rlip depletion by 2HF, Rlip antisense (R508), or Rlip shRNA could inhibit endocytosis in mouse melanoma cells. Using fluorescence microscopy (Figure 3 and Appendix A Figure A1), and fluorescence-activated cell sorting (FACS) (Figure 4), we found that 2HF was a highly effective inhibitor of endocytosis of fluorescently labeled EGF in B16-F0 and B16-F10 cells, with inhibitory effects similar to those observed by depletion of Rlip by RLIP76-antisense or shRNA. These results indicate that inhibition of endocytosis and induction of apoptosis by Rlip depletion is a possible mechanism of anticancer effects in mouse melanoma.

### 2.5. 2HF Treatment Inhibits Melanoma Signaling in Vitro

**Figure 5:**
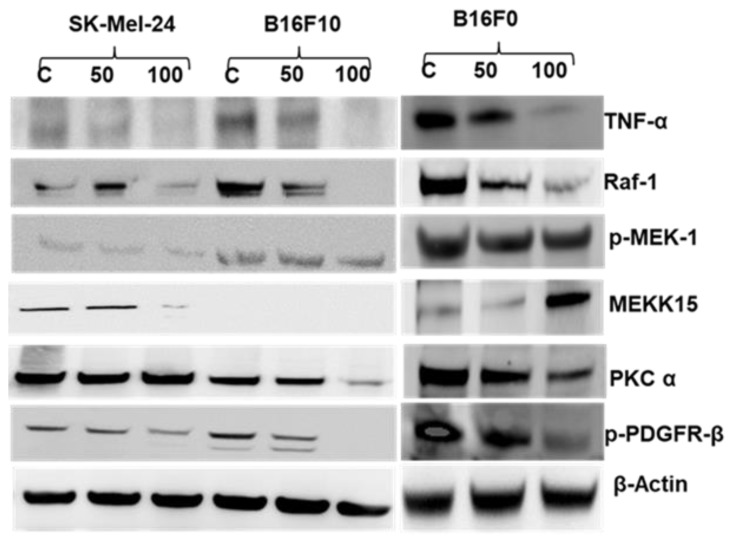
Effects of 2HF treatment on markers of intracellular signaling of mouse and human melanoma cells. Western blot analyses of signaling proteins in B16-F0, B16-F10, and SK-MEL-24 cell lysates in control and 50 or 100 µM 2HF-treated (48 h) experimental groups (*n* = 3).

To determine which signaling pathways might be involved in the 2HF action on melanoma cells, we tested its effects on signaling pathways known to be regulated by CDE, shown previously to be altered by Rlip-deficiency, or reported to cause treatment resistance in melanoma. PKCα is an overarching regulator of CDE for which Rlip is an essential effector [17]. We have shown that PKCα, MEKK-1, MEKK-15, TNFα, RAF1, and PDGFR-β are differentially regulated by Rlip deficiency (Supplemental Tables in [30]). In addition, Rlip targeting directly regulates AMPK [28,44]. Many human melanomas have mutations resulting in constitutively activated TNFα (inflammation response) [45,46], RAF-1 (CRAF proto-oncogene serine/threonine-protein kinase) [47], MEK/MAPK (mitogen-activated protein kinases) [48], PKC-α (protein kinase C alpha) [49], or PDGFR-β (platelet-derived growth factor receptor, beta polypeptide) [50]. As seen in Figure 5, treatment with 2HF at 50 or 100 µM reduced the expression levels of TNFα, RAF1, and p-PDGFR-β proteins, each of which has been implicated in causing treatment resistance in melanoma. 

MEKK-15 (also known as MAP3K-15, ASK-3, or apoptosis signal-regulating kinase-3) is a stress-responsive and pro-apoptotic atypical kinase of the JNK pathway upstream of MEK1 activated by reactive oxygen species [51]. Its importance in melanoma is not fully understood since it has been shown to be frequently lost in metastatic melanoma in females [52], but also to be upregulated in metastatic melanoma [53]. We found that MEKK-15 was undetectable in the high-metastatic potential B16-F10 cell line. 2HF increased the expression of this pro-apoptotic protein in the B16-F0 cells, which have much lower metastatic potential [54]. Because 2HF had the opposite effect on MEKK-15 level in the BRAF-mutated human SK-MEL-24, no firm conclusion could be drawn as to whether this kinase plays a role in the mechanisms of 2HF effects (Figure 5).

### 2.6. 2HF and Sunitinib Co-Treatment on Mouse and Human Melanoma Cells in Vitro

Sunitinib can be used for treatment of patients with BRAF wild-type melanoma [55], but is relatively ineffective, thus strategies to increase its effectiveness would improve treatment of approximately half of melanoma patients who lack BRAF mutations. Sunitinib inhibits cellular signaling by targeting multiple receptor tyrosine kinases and several receptors including vascular endothelial growth factor receptors (VEGFR-1, -2, and -3) and platelet-derived growth factor receptors (PDGFR-α and PDGFR-β) [23,56,57]. It reduces vessel density and induces hypoxia that is associated with the expression of VEGF-A in melanoma lines [58]. Rlip has also been shown to regulate angiogenesis through VEGF signaling [59].

**Figure 6:**
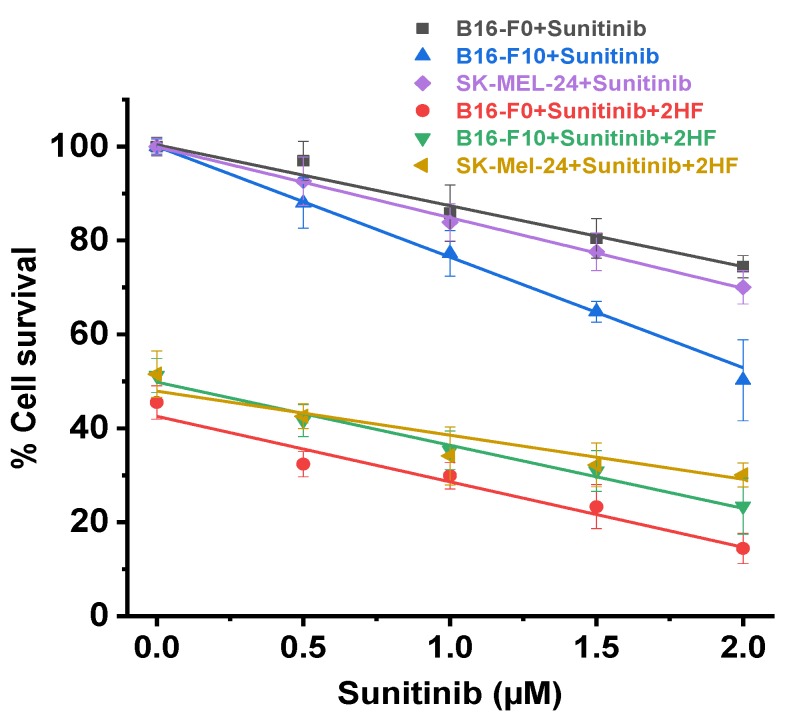
Mouse and human melanoma cell survival following sunitinib treatment with and without concomitant 2HF. Drug sensitivity assays were performed by MTT assay using 2HF at 72 h post-treatment to determine IC_50_. Cell proliferation assays were performed following sunitinib or 40 µM 2HF + sunitinib treatment for 72 h by MTT to determine additive effect of combination treatment. Values are presented as mean +SD from five separate determinations with eight replicates each (*n* = 5).

Because 2HF inhibited activation of PDGFR-β, we examined whether it would potentiate sunitinib, a kinase inhibitor active in both BRAF wild-type and mutant cell lines [33]. At each concentration of sunitinib (0.5, 1.0, 1.5, and 2.0 nM), the presence of 40 µM 2HF caused greater growth inhibition than without 2HF, with similar effects on all three cell lines. It is evident from similarly sloping curves (Figure 6) and Chou–Talalay analysis (combination index values between 0.9 and 1.1 for all cell lines) that the effects of 2HF and sunitinib were additive [60]. Though not synergistic, our results suggest that concomitant treatment with sunitinib and 2HF could substantially lower the required dose of sunitinib, potentially reducing sunitinib toxicity. This possibility will be studied in the future in animals, with subsequent clinical trials if animal studies confirm at least additive efficacy without increased toxicity.

### 2.7. 2HF is Poorly Orally Absorbed and Rapidly Metabolized after Oral or IV Dosing

**Figure 7:**
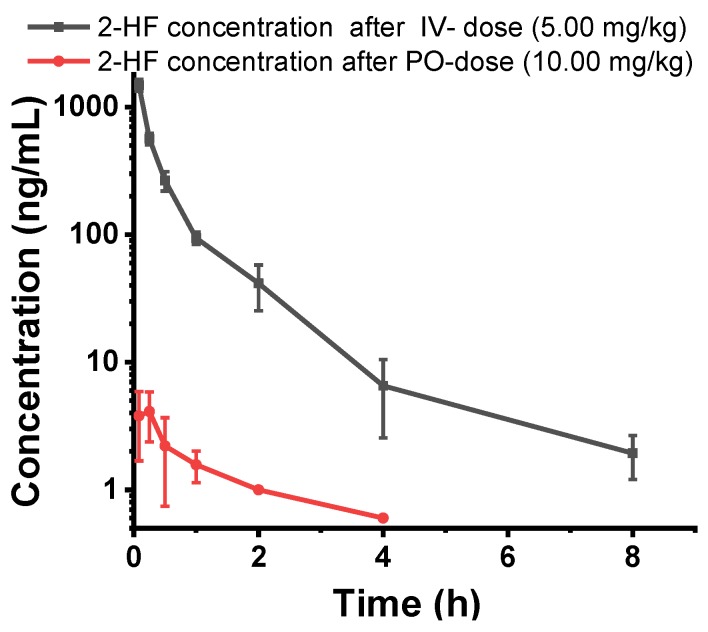
Plasma concentration vs. time profile of 2HF in rats. Mean plasma concentration vs. time profile of 2HF following intravenous and oral administration of 2HF at 5 mg/kg and 10 mg/kg, respectively, to male SD rats (*n* = 3).

Since our studies indicated that 2HF could be useful in the therapy of melanoma when used alone and in combination with sunitinib or PARP1 inhibitors, we considered whether it could be used as systemic therapy as an adjunct to these drugs. We have previously shown that 2HF dissolved in corn oil and given orally is an effective antineoplastic agent in animal studies. However, the pharmacology of 2HF without corn oil had not been studied. Thus, we performed an initial single-dose pharmacokinetic evaluation of oral (PO) and intravenous (IV) 2HF. At 24 h after administration, blood samples were collected, plasma concentrations of 2HF were measured, and its pharmacokinetic parameters were calculated. Poor oral bioavailability of 2HF was evident from two log-order lower plasma concentrations of 2HF when administered orally compared with IV. In addition, rapid clearance after either oral or IV administration suggested that 2HF would not be a good systemic agent for melanoma therapy without substantial pharmacological optimization (Figure 7). However, the excellent solubility of 2HF in PLO gel suggested that topical application may be a feasible and effective treatment.

### 2.8. Topical 2HF Inhibited the Growth of Implants of B16-F0 and B16-F10 Melanoma

**Figure 8:**
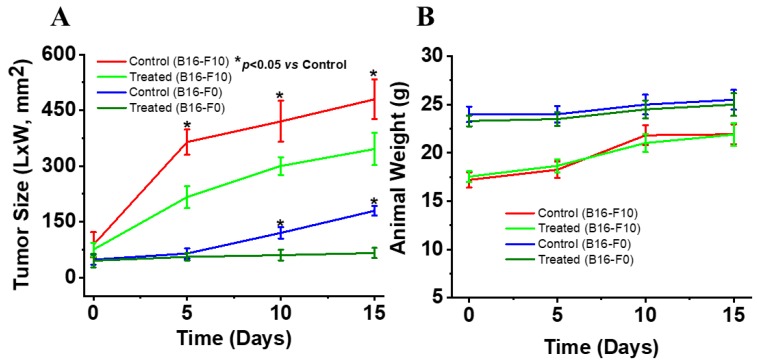
Anticancer effect and body weight changes with topical 2HF in mouse melanoma. (**A**) 2HF in Pluronic Lecithin Organogel (PLO) gel (12 mg/0.1 mL) was applied to the surface of B16-F0 melanoma subcutaneous syngeneic tumors and 20 mg/0.1 mL on B16-F10 melanoma (intradermal implant) C57BL6 mice (*n* = 8 per group). Treatment began on day 0 (10 days after subcutaneous/intradermal injection of 1 × 10^6^ cells/0.1 mL PBS), at which time all mice had palpable melanoma nodules ~ 8 × 8 mm in size. Bi-dimensional measurements were made using calipers and the product of these measurements was plotted vs. time (days). (**B**) The animal weights (g) as measured during the experiment are shown.

**Figure A2:**
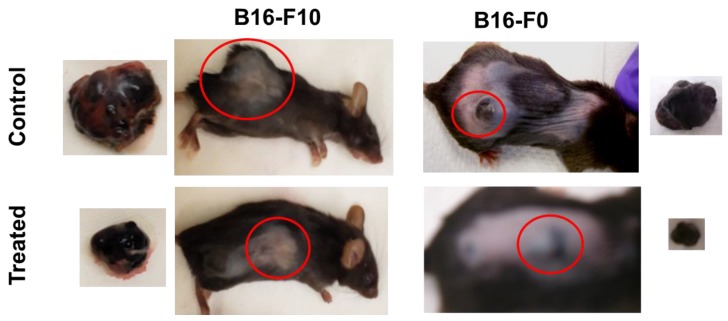
Photographs of the melanoma nodule of one representative mouse from each group are shown with red circle showing tumor. On day 15, the mice were euthanized. Excised tumors are presented with photographs at the same magnification of the excised tumor of another representative mouse from each group.

2HF is a potent anticancer agent that has been tested against various cancer types, but its efficacy against melanoma has not been previously established. Therefore, we tested the effect on melanoma in vivo upon 2HF topical application. Pluronic Lecithin Organogel (PLO) is a ready-to-use biphasic-compounding kit for PLO transdermal gel preparations. It consists of organic and aqueous phases and is compatible with a wide range of therapeutic agents. It utilizes a thermodynamically stable and pharmaceutically acceptable transdermal drug delivery system. We used 2HF–PLO preparations in two sequential experiments using syngeneic B16-F0 and B16-F10 melanoma-bearing mice (*n* = 8 for each group of treatment). Results of these studies demonstrated that the topical application of 2HF in PLO gel caused a dose-dependent growth inhibition of subcutaneously (B16-F0) or intradermally (B16-F10) implanted mouse melanoma tumors in immunocompetent mice (Figure 8A; Appendix A Figure A2) without affecting normal weight gain (Figure 8B).

### 2.9. 2HF Regulates Cancer Signaling in Tumors Induced by B16-F0 and B16-F10 Melanoma Cells

**Figure 9:**
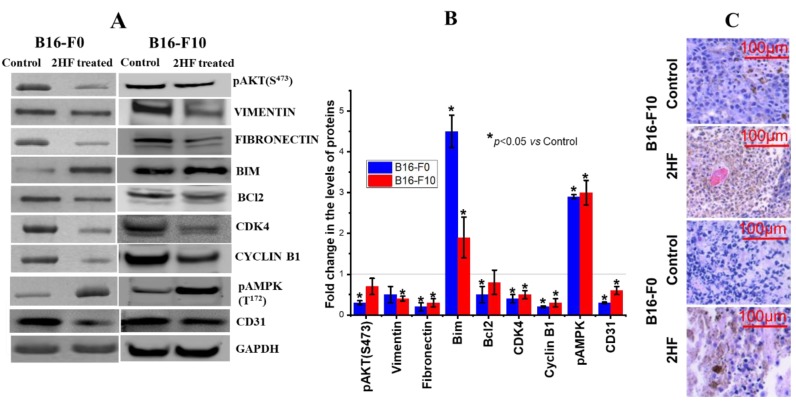
Effects of 2HF treatment on markers of intracellular signaling in tumors. Western blot analyses of signaling proteins in tumor tissue lysates in control and topically 2HF in PLO gel (12 or 20 mg/0.1 mL)-treated experimental groups (**A**). The bar diagrams represent the fold change in the levels of proteins as compared to control (dotted line) as determined by densitometry (*n* = 4) (**B**). Photomicrographs of hematoxylin and eosin (H&E) staining of tumor sections. Control H&E-stained sections of dissected tumors show the intact tumor cell structure. Cells have distinct nuclei with a thin uniform cytoplasmic rim around each individual cell. Tumors treated with 2HF show a non-uniform thickened rim of cytoplasm around the damaged nuclei (**C**).

The tumors removed from 2HF-treated and control mice were examined by routine histology on hematoxylin and eosin (H&E) stained slides and Western blot. Increased p-AMPK and decreased CD31 expression were confirmed in tumor tissue from Western blot analyses and the corresponding densitometry quantitation (Figure 9A,B). Western blot analyses demonstrated reduced activation (pAKT) of the survival signal AKT in B16-F0 tumors. Cell cycling proteins CDK4 and cyclin B1 were also reduced, as were markers for epithelial–mesenchymal transition (EMT) and fibronectin in both tumor types but vimentin was reduced in tumors induced by B16-F10. The pro-apoptotic protein BIM was increased in both models, and the anti-apoptotic protein BCL2 was reduced in B16-F0 (Figure 9A,B). Importantly, these signaling pathways are regulated by endocytosis of the upstream ligand–receptor complexes [22,25,30,61,62]. H&E-stained sections of dissected control and treated tumors show an increase in apoptotic nuclei in treated groups (Figure 9C).

### 2.10. 2HF Was Absorbed Systemically but Did Not Exhibit any Significant Systemic Toxicity

The skin barrier represents a major challenge pertaining to cutaneous administration of drugs in clinical practice. Therefore, we determined the systemic absorption of 2HF into serum using LC-MS/MS and quantified it using a standard curve of 2HF. There was significant systemic absorption shown by measurements of serum levels of 2HF. LC-MS/MS analysis of serum from 2HF-treated (topical application of 12 mg 2HF/0.1 mL) mice revealed that 2HF was absorbed after topical application reaching 4.3 ± 0.4 µM in serum. This level of systemic absorption of 2HF caused no adverse effects on blood counts, renal function, or liver enzymes. Interestingly, as with Rlip depletion [33], even low levels of serum 2HF were associated with significantly lower triglycerides and cholesterol (*p* < 0.05). LDH was also notably decreased in the 2HF group, consistent with tumor shrinkage and disease control (Table 2). Although statistically significant changes in alanine transaminase (ALT), aspartate transaminase (AST), and alkaline phosphatase (ALP) levels occurred in the experimental group, all values remained within the normal range.

## 3. Discussion

We demonstrate, for the first time, that 2HF exhibits significant antineoplastic activity towards murine and human melanoma cell lines and that topically applied 2HF–PLO gel can reduce the growth of subcutaneously or intradermally implanted mouse melanoma in immunocompetent mice. 2HF broadly inhibited cancer-promoting signaling in vitro and in vivo in B16-F0 and B16-F10 cells.

TNF-α, PKC-α, fibronectin, CDK4, cyclin B1, and CD31were inhibited by 2HF, while BIM and pAMPK were activated. The pattern of signaling inhibition in melanoma upon treatment with 2HF is similar to that observed previously upon Rlip inhibition or depletion in renal cell carcinoma, breast cancer, neuroblastoma, prostate cancer, pancreatic adenocarcinoma, non-small cell and small cell lung carcinoma, and lymphoma [23,25,26,31,63,64,65,66]. This finding strengthens the assertion that 2HF inhibits melanoma growth through Rlip inhibition.

The signaling effects on TNFα, RAF-1, and PDGFR-β as well as the cytotoxicity of 2HF was similar among all three cell lines. Nearly half of patients with cutaneous and nearly all patients with uveal melanomas lack BRAF mutations, thus are not candidates for highly effective therapy with BRAF/MEK inhibitor combinations. Their therapeutic options are further limited because BRAF-wild-type melanomas frequently display treatment resistance due to activated MEK1 (MAP2K1), PKCα, PDGFR-β, AKT, or TNFα pathways. Sunitinib, a broad-spectrum tyrosine kinase inhibitor that primarily targets VEGF pathways but has some collateral inhibition of these pathways, can be used for BRAF-wild-type or drug-resistant BRAF-mutant melanomas, but response rates are low and toxicity is significant [50,56]. Our results suggest that 2HF could potentiate the efficacy of sunitinib in these patients and perhaps allow for a reduction in sunitinib dose to reduce toxicity.

The global effects on melanoma signaling are consistent with Rlip being an important component of clathrin-dependent endocytosis (CDE) [17]. It is bound to the clathrin coat through the AP2 adaptor protein and is thought to provide energy for CDE through ATP hydrolysis that is coupled to efflux of GS-E [17]. Formation of the Rlip/Clathrin/AP2/POB1 signalosome allows for the formation of vesicles and the endocytosis of activated EGF receptors [41,42], and Rlip-null mouse embryonic fibroblasts (MEFs) are deficient in CDE [30]. We have also shown that the rate of GS-E efflux by Rlip mutants correlates with apoptotic activity and with the rate of EGF and insulin receptor endocytosis [17]. 2HF induces regression of renal cell carcinoma xenografts and causes signaling effects similar to those observed with Rlip-specific antisense, siRNA, or anti-Rlip antibodies [23,43]. Additionally, we have reported in lung cancer that 2HF directly binds to Rlip, inhibiting its transport functions and potentiating the anticancer effects of anti-Rlip antibodies, indicating that Rlip inhibition is a mechanism of 2HF activity [14].

BRAF wild-type melanomas frequently carry mutations in the NRAS or p53 genes. Cancers mutated in p53 or NRAS are known to be notoriously resistant to nearly all types of therapies, and therefore treatments that can overcome the treatment resistance conferred by these mutations are of fundamental importance in cancer therapy. In the present study as well as studies of multiple other types of cancer, we have shown that 2HF or Rlip depletion by antisense or antibody exerts antineoplastic effects in p53 wild-type or mutated cancers. The importance of Rlip targeting by 2HF is significant and impactful in light of our recent studies establishing an existential requirement for Rlip in p53-null malignancy [30]. We found that haploinsufficiency of Rlip completely switched off spontaneous malignancy in p53-null mice that always develop malignancy otherwise, and that this was accompanied by nearly complete normalization of the epigenome and transcriptome [30].

Interestingly, our studies suggest that PARP1 inhibitor activity may be enhanced by 2HF. At a higher concentration, 2HF significantly reduced expression of PARP1 in all three cell lines tested here. It is of interest that SK-MEL-24 was susceptible to this effect, implying that 2HF could also increase the effectiveness of PARP1 inhibitors in melanomas that are deficient in non-homologous end joining (NHEJ) DNA-repair. A recently published study showed potentiation of the cytotoxic effect of alkylating agents on melanoma cells by addition of a PARP1 inhibitor [34,35,36]. Furthermore, 20 µM 2HF reduced the IC_50_ of PARP1 inhibitor in B16-F0 and B16-F10 melanoma cells, but the combination effect on SK-MEL-24 was not statistically significant.

Systemic absorption of 2HF caused no overt toxicity. The concentrations of 2HF necessary to inhibit the growth of melanoma cells were relatively high (µM) when compared with existing drugs, which are generally effective in nM range. Though this implies the necessity for developing pharmacologically optimized derivatives, the non-toxic nature of 2HF suggests that a suitable oral or IV formulations could be useful as a systemic therapy. In contrast, very high local concentrations of 2HF (mM) would be expected locally upon local application. Thus, 2HF applied topically in a suitable formulation could be translated to topical therapy of cutaneous metastases of melanoma even without further structural optimization. Its abundance in nature will certainly facilitate its clinical translation and the drug product would be expected to be relatively inexpensive and non-toxic. Given that 2HF has very similar in vitro IC_50_ values towards, lung, breast, and renal cancer, it is certainly conceivable that topical 2HF could be effective as a topical therapy for cutaneous metastases from these cancers as well.

In summary, our studies show that a 2HF–PLO gel is active when applied topically to cutaneous melanoma, that the mechanism of action appears to be through Rlip protein depletion, and that it may enhance the anticancer effects of sunitinib and PARP1 inhibitor. Our studies provide a strong rationale to develop 2HF for topical therapy of skin metastases of melanoma.

## 4. Materials and Methods

### 4.1. Reagents and Cell Lines

3-(4,5-dimethylthiazol-2-yl)-2,5-diphenyltetrazolium bromide (MTT), dimethyl sulfoxide (DMSO), and 2HF were obtained from Sigma (St Louis, MO, USA). Pierce™ Clear Milk Blocking Buffer (10×) was purchased from ThermoFisher Scientific (Waltham, MA, USA). CD31, Ki67, cyclin B1, CDK4, cleaved PARP, vimentin, AKT, pAKT (S^473^), fibronectin, BIM, BCL2, p-AMPK(T^172^), TNFα, RAF-1, p-MEK, MEKK, PKC, p-PKCα, p-PDGFR, beta-actin, and GAPDH antibodies were purchased from Santa Cruz Biotechnology (Columbus, OH, USA). TUNEL fluorescence and avidin/biotin complex (ABC) detection kits were purchased from Promega (Madison, WI, USA) and Vector (Burlingame, CA, USA), respectively. AZD 2461 (PARP1 inhibitor) was purchased from Bio-Techne Corporation (Minneapolis, MN, USA), and Quick apoptotic DNA ladder kit was purchased from Life Technologies (Carlsbad, CA, USA). The mouse B16-F0, B16-F10, and human SK-MEL-24 cell lines were purchased from the American Type Culture Collection (ATCC, Manassas, VA, USA). Cell lines were cultured in vitro in 10% FBS-supplemented Dulbecco’s Modified Eagle’s Medium (DMEM). 2HF for in vitro dosing was prepared at 20 mM in methanol. Recombinant murine EGF was purchased from Peprotech (Rocky Hill, NJ, USA). NHS-rhodamine, pHrodo™ Red Epidermal Growth Factor (EGF) Conjugate, and Lipofectamine 2000 were purchased from Thermo Fisher (Waltham, MA, USA). RLIP76 shRNA plasmid DNA and respective controls were a kind gift from Dr. Lawrence E. Goldfinger (Thomas Jefferson University, Philadelphia, PA, USA). Chemically synthesized phosphorothioate DNA R508 (5′-GGCTCCTGAATTGGCTTTTTC-3′) in desalted form was purchased from Biosynthesis, Inc., (Lewisville, TX, USA). A 21-nucleotide-long scrambled phosphorothioate DNA CAS (5′-GTTATTGCTTCGTTCCGCGAT-3) was used as a control [30]. A ready to-use biphasic compounding kit (from a local pharmacy) for pluronic lecithin (PLO) transdermal gel preparation, consisting of organic and aqueous phases, was used for topical 2HF delivery system [67]. All other reagents used in this study were analytical grade.

### 4.2. Ethics Statement

The work on B16-F0 melanoma in normal C57BL/6 mice was performed in the City of Hope National Medical Center (COHNMC), Duarte, CA, USA and work on B16-F10 melanoma in normal C57BL/6 mice was performed in Texas Tech Health Sciences Center (TTUHSC), Lubbock, TX. The Institutional Animal Care and Use Committees of COHNMC, Duarte, CA, USA and TTUHSC, Lubbock, TX, USA approved all animal protocols (protocol code: 17020). Animals were housed in the respective animal care units. A pilot study to establish the dose, frequency, and duration of 2HF topical application required for tumor regression in wild type (WT, C57BL6) mice was performed, and an effective dose of 12 mg 2HF/0.1 mL for B16-F0 and 20 mg 2HF/0.1 mL for B16-F10 for 5 days/week was found to be effective. All procedures and experiments complied with their guidelines to minimize animal suffering.

### 4.3. IC_50_ of 2HF on Melanoma Cell Line

The IC_50_ of 2HF on B16-F0, B16-F10, and SK-MEL-24 cells was determined by MTT cytotoxicity assay. Cells were harvested and Trypan Blue dye exclusion test was used to determine the number of viable cells present in a cell suspension using a hemocytometer. The cell suspension was diluted to 5 × 10^4^ cells/mL with Dulbecco’s Modified Eagle’s Medium (DMEM) complete medium and 5000 cells/well were plated in 96-well plates. After 24 h incubation at 37 °C, cells were treated with 2HF concentrations ranging from 0–200 µM (8 replicates per plate). After 48 or 72 h incubation, 40 µL of MTT (5 mg/mL) solution was added. After 4 h incubation, the plate was centrifuged at 700× g for 5 min and medium was aspirated completely. Then, 50 µL DMSO was added to each well to dissolve the purple precipitate on the rocking platform for about 15 min. The absorbance was then measured at 490 nm using a 96-well microplate reader (Spectramax Plus, Molecular Devices, Inc, San Jose, CA, USA).

### 4.4. Effect of Sunitinib on 2HF-Mediated B16-F0, B16-F10, and SK-MEL-24 Cell Death

2-HF was dosed from 0–150 µM to determine the IC_50_ of 2HF. To determine how sunitinib co-treatment effects on the ability of 2HF to inhibit B16-F0, B16-F10, and SK-Mel-24 cell growth, 0–2.0 µM sunitinib was dosed alone and with a steady state background of 2HF dosed at IC_50._ Analysis was by MTT cytotoxicity assay as described above in Section 4.3.

### 4.5. Effect of 2HF on Key Signaling and Apoptotic Proteins Responsible for Melanoma Cell Growth/Death

B16-F0, F16-F10, and SK-MEL-24 cells were treated for 24 h with vehicle, 50 µM, and 100 µM 2HF. Cells were harvested and lysates prepared for Western blot using a radioimmunoprecipitation assay (RIPA) buffer with a protease (complete ULTRA) and phosphatase inhibitor (PhosSTOP) cocktail (Sigma-Aldrich, St. Louis, MO, USA). Total cell lysates (25 µg/lane) were loaded on 4–12% bis-tris gels, with MES gel running buffer. Proteins were transferred to nitrocellulose membrane, and nonspecific binding was minimized with 1× clear milk blocking buffer +0.1%TWEEN 20 (ThermoFisher Scientific, Waltham, MA, USA) for 1 h at room temperature. All primary antibodies were purchased from Santa Cruz Biotechnology except mouse monoclonal, TNF-α (Invitrogen), (1:1000), in 1× clear milk +0.1% TWEEN 20 overnight at 4 °C. Secondary antibody from Santa Cruz Biotech (1:2000) was incubated for 1 h at room temperature in 1× clear milk, 0.1% TWEEN 20.

### 4.6. Detection of 2HF-Induced Apoptosis in Mouse and Human Melanoma Cells by DNA Fragmentation

A total of 2.2 × 10^6^ B16-F0, B16-F10, or SK-MEL-24 cells were plated in four 100 mm dishes and incubated overnight. Each plate was treated with 40 µM of 2HF and incubated for 0 h, 24 h, 48 h, or 72 h at 37 °C. After incubation, cells from each dish were harvested and rinsed with phosphate-buffered saline (PBS), followed by their collection as pellets in micro tubes. DNA was extracted from cell pellets using the Quick Apoptotic DNA ladder detection kit and run on 1.2% agarose gels containing 1 μL/100 mL SYBR-Safe DNA gel stain (Invitrogen) at 50 volts for about 2 h. The gels were examined and photographed with a Bio-Rad gel documentation system (Gel Doc XR, Hercules, CA, USA).

### 4.7. Detection of 2HF-Induced Apoptosis in Mouse and Human Melanoma Cells by TUNEL Assay

A terminal deoxynucleotidyl transferase dUTP-mediated nick-end labeling (TUNEL) assay was utilized to assess and validate apoptotic cell death. TUNEL staining for FACS analysis was performed using an APO-BrdU TUNEL assay kit (Invitrogen, ThermoFisher Scientific), following the manufacturer’s protocol. Briefly, 2 × 10^5^ cells in 100 mm tissue culture dishes were grown in growth medium up to 70–80% confluency. Treatments with 50 and 100 µM of 2HF were performed as above. After required treatments, cells were washed with PBS and trypsinized. Cells (1–2 × 10^6^) were suspended in 0.5 mL PBS and added to freshly prepared 1% (*w*/*v*), buffered paraformaldehyde, and placed on ice. After 15 min, cells were washed twice with PBS, and ice-cold 70% (*v*/*v*) ethanol was added and kept on ice for 30 min. Cells were washed twice with wash buffer (provided with kit), resuspended in 50 µL of DNA-labeling solution, and incubated for 60 min at 37 °C in a water bath. Cells were rinsed two times with rinse buffer (provided with kit). Cell pellets were resuspended in 100 µL of antibody solution and further incubated for 30 min at room temperature in the dark. After incubation, cells were analyzed with the BD Accuri C6 Flow Cytometer (BD Biosciences, San Jose, CA, USA). The fluorescence level for discrimination between apoptotic and nonapoptotic cells was set using the control without TdT (terminal deoxynucleotidyl transferase). Cells above this fluorescence value in the TdT-positive sample were considered apoptotic. The percentages of cells undergoing apoptosis were assessed. Analysis was performed using the BD CSampler software (BD Biosciences). Data are shown as a logarithmic histogram and expressed as fluorescence intensity of number of counts of the TUNEL-positive cells obtained from the statistical analysis of the fluorescence height and mean value of the x-axis displayed by the software. Data were obtained from flow cytometry analyses from three independent experiments.

### 4.8. Effect of RLIP76 Depletion on EGF Binding and Internalization

The effect of Rlip knockdown/depletion by shRNA, RLIP76-antisense (R508), or 2HF on endocytosis was studied in B16 melanoma cell lines. The cells were transfected with Rlip shRNA or R508 antisense and their scrambled shRNA and scrambled antisense (CAS) controls as previously described [13,64]. Briefly, B16 cells were grown in DMEM medium containing 10% FBS on Nunc 4 chamber slides overnight to 70–80% confluence. For RLIP76 knockdown, pSUPER retro puro shRLIP plasmid DNA (8 µg/mL) and RLIP76-antisense (R508; 10 μg/mL final conc.) and their respective controls (scrambled shRNA and CAS) were pre-complexed with Lipofectamine 2000 reagent in Opti-MEM^®^ reduced serum medium for transfection of the cells. Other sets of slides were treated with vehicle (Methanol) or 10 µM 2HF to inhibit Rlip expression. After 24 h, cells were washed with PBS 4–5 times and incubated with 40 ng/mL EGF-rhodamine (prepared in PBS containing 1% BSA) [17,68,69] for 60 min in ice (4 °C) or EGF-pHrodo according to the manufacturer’s instructions. Cells were then incubated at 37 °C in a humidified chamber for 10 min followed by fixation with 4% paraformaldehyde. Slides were analyzed using a fluorescence microscope (Olympus America, Melville, NY, USA). Photographs taken at identical exposure settings at 200× magnification are presented.

The effect of Rlip knockdown on endocytosis was further checked by flow cytometry. For flow cytometry analysis, cells were grown on 60 mm tissue culture dishes. Rlip knockdown using RLIP76-antisense or Rlip shRNA or by treatment with 2HF was performed as described above. After 24 h, cells were trypsinized, washed with cold PBS, and counted. Cell samples (5 × 10^5^ to 1 × 10^6^ cells) were incubated on ice with sample blocking buffer (10% goat serum and 1% bovine serum albumin) for 20 min, after which samples were centrifuged at 200× g for 5 min, followed by washing with cell staining buffer (1% BSA in PBS). Cells were then incubated with perm buffer (BD Biosciences) for 10 min on ice. After washing 3 times with PBS, cells were then incubated with EGF-pHrodo complex on ice for 45 min, washed with cold cell staining buffer three times, and incubated for 10 min at 37 °C. Cells were washed with cell staining buffer 3 times, then resuspended in 0.2 mL of staining solution. Acquisition and analysis were performed on LSRFortessa flow cytometer (Becton, Dickinson and Co., Franklin Lakes, NJ, USA), using FACS Diva software v8.0.1 (Becton, Dickinson and Co., Franklin Lakes, NJ, USA). Viable cells were identified by gating on forward and side scatters (FSC/SSC, representing the distribution of cells in the light scatter based on size and intracellular composition respectively). At least 10,000 cells were analyzed per staining. Data are shown as logarithmic dot plots and expressed as mean fluorescence intensity, obtained from the statistical analysis of the fluorescence height and mean value of the x axis displayed by the software. Data were obtained from three independent experiments.

### 4.9. Animal Studies

C57BL/6 mice were obtained from Harlan, Indianapolis, IN. Two sequential experiments each testing a different concentration of 2HF (12 mg 2HF/0.1 mL and 20 mg 2HF/0.1 mL) against placebo were performed to test the efficacy of topical 2HF against melanoma cells in C57BL6 mice. Each experiment tested twelve 11–12-week-old C57BL6 mice (6/group) that were injected in one flank with 1 × 10^6^ mouse B16-F0 (subcutaneously) or B16-F10 (intradermal) melanoma cells in 0.1 mL PBS. At 10 days after injection of melanoma cells, the mice developed palpable tumors at least 8 × 8 mm in size. After randomization to treatment groups, mice were treated with once daily topical application of 0.1 mL PLO (pluronic lecithin organogel) alone or with PLO containing either 12 or 20 mg 2HF/0.1 mL PLO gel. The mice were examined daily for signs of distress and tumor growth. Tumors were measured in two dimensions using calipers. On day 15 from the start of treatment, the mice were euthanized. The average (*n* = 8/group) product of maximal perpendicular diameters was plotted against days.

### 4.10. Assessment of Angiogenesis, Proliferation, and Apoptosis

B16 melanoma tumors from experimental and control arms were harvested from mice and used for histopathologic analyses. Tumor samples fixed in buffered formalin for 12 h were processed conventionally for paraffin-embedded tumor sections (5 µm thick). Hematoxylin and eosin (H&E) staining was performed on paraffin-embedded tumor sections. Western blot analysis was performed by separating control and treated tumor lysates on SDS-PAGE gels followed by transfer to nitrocellulose membrane. Expression levels of pAKT, AKT vimentin, fibronectin, BIM, BCL2, CDK4, CD31, cyclinB1, pAMPK, and GAPDH were determined using corresponding antibodies.

### 4.11. LC-MS/MS Analysis of 2HF in Serum

C57BL/6 mice were administered either 0.1 mL PLO gel or 12 mg 2HF in 0.1 mL PLO gel topically each day for 8 weeks. On the last day, the blood was collected within 2 h after final dosage, and subjected to LC-MS/MS analyses according to established protocol. Briefly, serum from control and 2HF-treated mice was extracted twice with 500 µL ethyl acetate and the organic layers were collected and evaporated to dryness in a speed-vac. The residue was reconstituted in 40 µL methanol containing 0.1% formic acid (FA) for analysis. An LC-MS method was developed for the quantitative analysis of 2HF using an Agilent 6490 triple quadrupole coupled with an Agilent 1290 UHPLC system. In short, the binary pump delivered buffers A (0.1% FA in water) and buffer B (0.1% FA in acetonitrile) over 10 min with the following gradient: From 10% B to 20% B in 1 min, then ramp to 90% B at 6 min, 10% B at 8 min, 10% B at 8.5 min, column equilibration post analysis was 2 min. An Agilent Zorbax, 0.5 × 150 mm, 5 µm particle size was used for the separation at a flow rate of 50 µL/min.

### 4.12. Single Dose Pharmacokinetic Study of 2HF

Pharmacokinetic studies of 2HF were performed by GVK Biosciences Pvt. Ltd., Hyderabad, India in Sprague Dawley male rats (~280 g). Rats were dosed either orally through gastric gavage needle of 10 mg/kg 2HF (0.5% Methyl cellulose + 0.5% Tween80) or intravenous 5 mg/kg 2HF (in 10% DMSO). Then, 200–300 µL blood was collected at each time point and plasma was separated; 50 µL plasma sample was deproteinized with 200 µL of acetonitrile followed by centrifugation at 2000× g for 5 min and supernatant was analyzed for 2HF by LC-MS/MS. Pharmacokinetic parameters were calculated for individual animals by a non-compartmental model with Phoenix software version 8.1 (CERTARA, Princeton, NJ, USA).

### 4.13. Effect of 2HF on Blood Chemistry of B16-F0 Melanoma-Bearing C57B Mice

Blood samples were collected from representative mice from the control and 2HF-treated with 12 mg/0.1 mL PLO gel topically each day for 8 weeks just before euthanasia. These samples were used for hematologic, hepatic, renal, and metabolic profiling. A Hemavet instrument (Drew Scientific, Dallas, TX, USA) was used for determination of hematologic parameters.

### 4.14. Statistical Analysis

All data comparing two groups were evaluated with a two-tailed unpaired student’s *t* test are expressed as the mean ±SD. Changes in tumor size and body weight during the course of the experiments were visualized by scatter plot. The statistical significance of differences between control and multiple treatment groups was determined by ANOVA followed by multiple comparison tests. Differences were considered statistically significant when the *z-*score was either above 2 or below −2, and the *p* value was less than 0.05.

## 5. Conclusions

Our studies demonstrate, for the first time, that 2-HF, a non-toxic compound abundant in orange peel, can reduce the growth of melanoma when a PLO gel containing 2HF is applied topically. We show that 2HF inactivates multiple signaling mechanisms that support the growth and survival of melanoma and play central roles in mediating resistance to kinase inhibitor and immunotherapy. Evidence presented here supports a mechanistic model in which 2HF exerts its anticancer activity through targeting Rlip, resulting in inhibition of clathrin-dependent endocytosis, thus attenuating several key pathways that promote melanoma growth and resistance. Given the lack of observed toxicity in the present studies, topical 2HF appears to be an attractive approach for treatment of the intractable problem of cutaneous metastases of melanoma, and perhaps other malignancies. The activities of systemic kinase inhibitor and checkpoint inhibitor therapies could also be enhanced by topical 2HF.

## Figures and Tables

**Table 1:** Effect of 2HF on melanoma cell proliferation is independent of Breast Cancer gene (BRCA) expression status.

Parameter	B16-F0	B16-F10	SK-MEL-24
2HF IC_50_	38.59 ± 1.61 µM	41.95 ± 1.83 µM	44.92 ± 1.67 µM
PARP1i IC_50_	16.52 ± 4.86 µM	29.88 ± 28.53 µM	55.00 ± 23.90 µM
PARP1i IC_50_ with 20µM 2HF	10.31 ± 1.41 µM	12.86 ± 2.72 µM	43.02 ± 16.21 µM
Intact PARP1	++	+	++++
Cleaved PARP1 C-terminus	+	++	+++
Cleaved PARP1 N-terminus	+++	++	++
BRCA1 detection by WB	+	-	-
BRCA2 detection by WB	-	-	+

**Table 2:** Effect of 2HF (12 mg/0.1 mL PLO gel) on blood counts and serum chemistry measurements.

Parameter	Vehicle (PLO Gel)	2HF + PLO Gel Treated (Topically Each Day)
**CBC**		
RBC (×10^6^/µL)	8.6 ± 0.3	8.7 ± 0.2
WBC (×10^3^/µL)	9.0 ± 0.7	9.4 ± 0.7
Platelets (×10^3^/µL)	746.0 ± 48	689.0 ± 54
Hemoglobin (g/dL)	13.6 ± 0.3	13.7 ± 0.4
Hematocrit (%)	42.4 ± 0.8	41.8 ± 1.1
**Plasma/Serum**		
Glucose (mg/dL)	176.0 ± 12.4	179.0 ± 15.7
Creatinine (mg/dL)	0.17 ± 0.0	0.19 ± 0.0
Albumin (g/dL)	2.3 ± 0.1	2.8 ± 0.1
ALT(units/L)	105.0 ± 8.7	84.0 ± 5.4 *
AST(units/L)	414.0 ± 14.2	311.0 ± 12.0 *
ALP(units/L)	75.7 ± 4.6	108.4 ± 7.2 *
LDH(units/L)	6126.0 ± 274.0	2150.0 ± 123.0 *
Triglycerides(mg/dL)	166.0 ± 9.7	111.0 ± 7.3 *
Cholesterol (mg/dL)	98.0 ± 5.5	83.0 ± 2.4
*n* = 3 mice in each group * *p* < 0.05		

## Supplementary Materials

Supplementary File 1
