# Supplementary material for: Topical 2′-Hydroxyflavanone for Cutaneous Melanoma
Source: Cancers (Basel). 2019 Oct 14;11(10):1556. doi: 10.3390/cancers11101556 (PMC6826616; doi:10.3390/cancers11101556)

Supplemental Figure 1

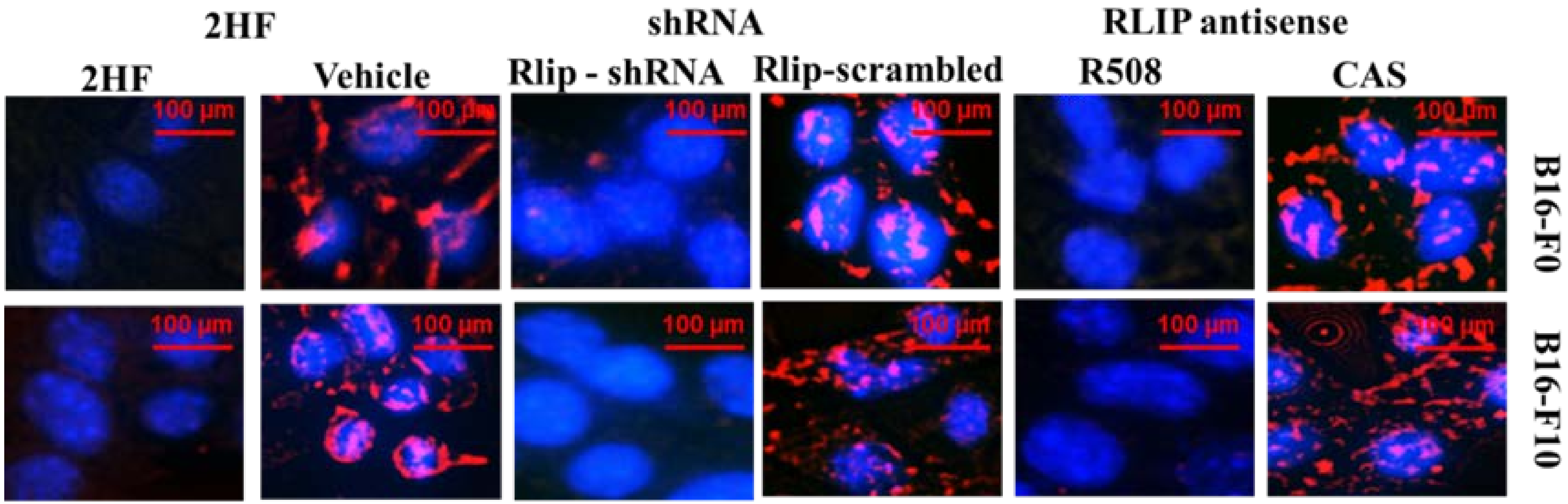

Supplemental Figure 2

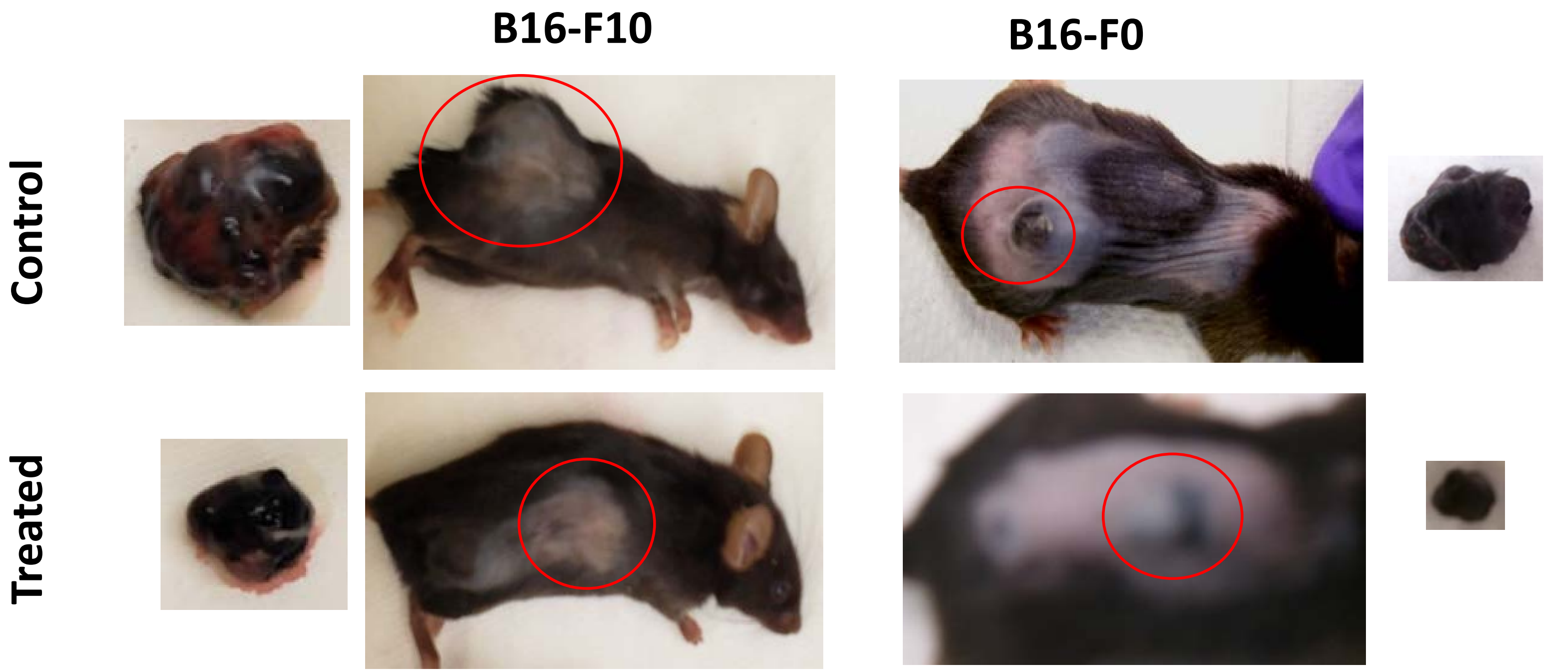

Supplemental Figure 3

Supplemental data for Fig. 2C.

Numbers below of each blot represent protein band intensity ratios to adequate controls after normalization against **β-Actin**. **Full blots are in next 3 figures.**

RAL BP-1 and apoptosis enzymes in SK-Mel-24, B16F0 and B16F10 cells after treatment with 50, 100 µM 2HF for 48h. Cells were plated in complete growth medium. After overnight, cells were treated with different doses of 2HF, and incubated for 48h. Total cell lysates (40 µg/lane) were loaded on 4-12% bis-tris gel, with 1X MES gel running buffer. Proteins were transferred to nitrocellulose membrane, nonspecific binding was done in 1X clear milk, +0.1%T20 (Invitrogen) for 1h at room temperature. All primary antibodies were from (mouse monoclonal, Santacruze Biotechnology (1:1000), in 1X clear milk +0.1%T20 overnight in 40C. Secondary antibody from Santacruze Biotech (1:2000) for 1h at room temperature in 1X clear milk, 0.1% T20.

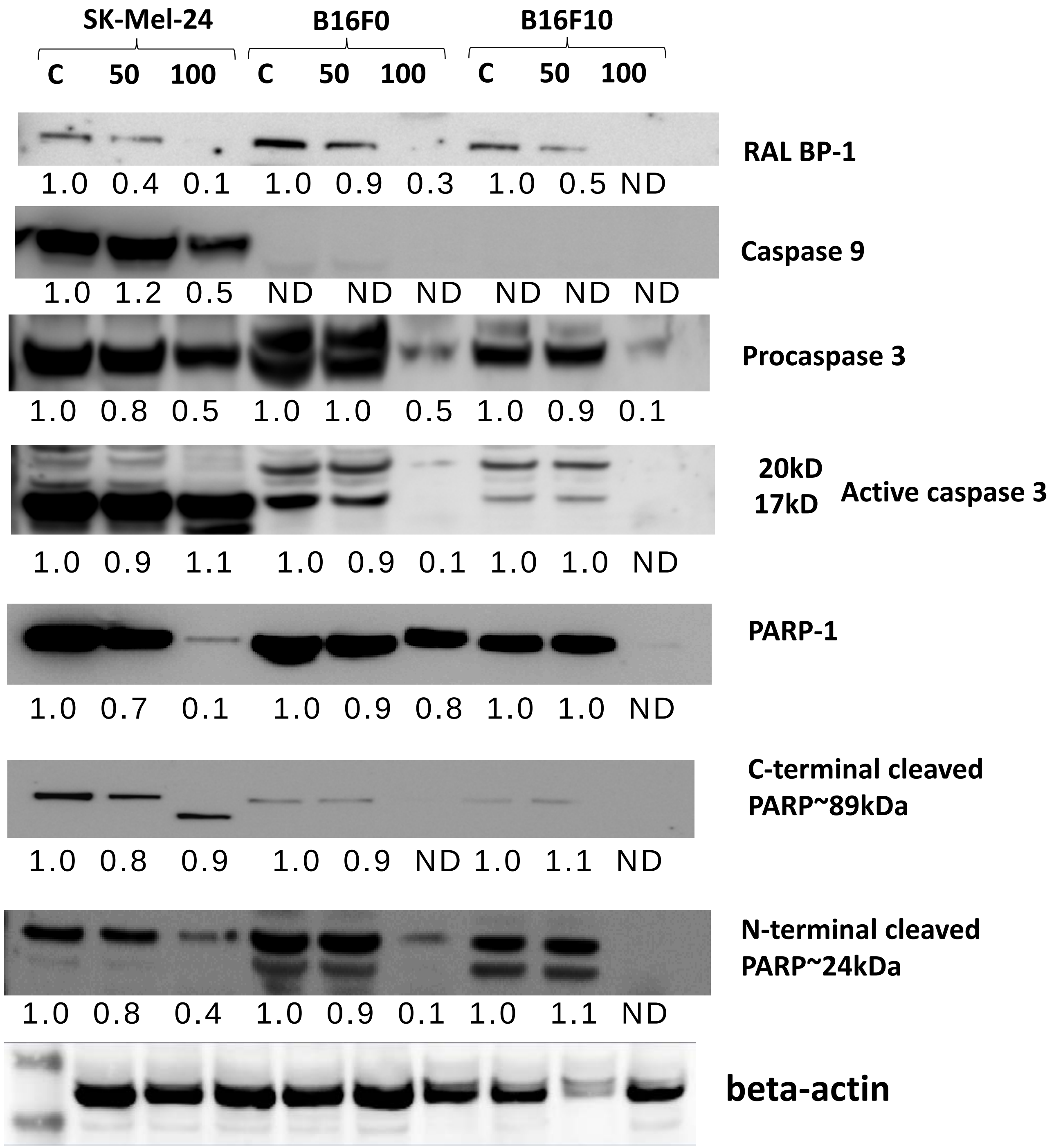

Supplemental Figure 4

Supplemental data for Figure 2C. Effect of 2HF on B16-F0, B16-F10 and SK-MEL-24 cells. Representative unprocessed western blots of control and 2HF treated animal tumors as shown in figure 9A. Red Arrow show cropped area included in figure 9A. Light blue arrows show molecular weight markers.

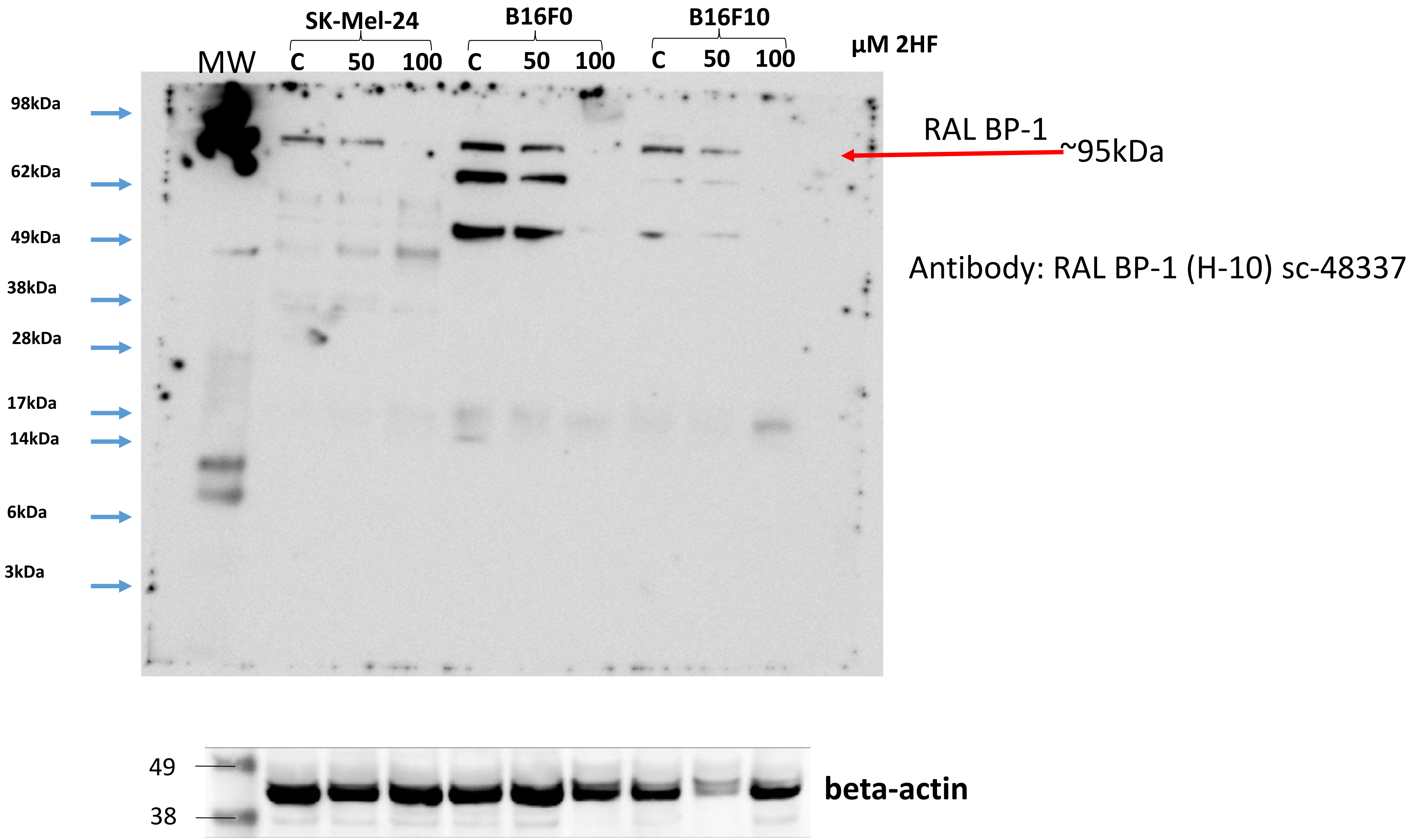

Supplemental Figure 5

Supplemental data for Figure 2C. Effect of 2HF on B16-F0, B16-F10 and SK-MEL-24 cells. Representative unprocessed western blots of control and 2HF treated animal tumors as shown in figure 9A. Red Arrow show cropped area included in figure 9A. Light blue arrows show molecular weight markers.

Antibody: Caspase-9 p10(F-7):sc-17784

Caspase-9

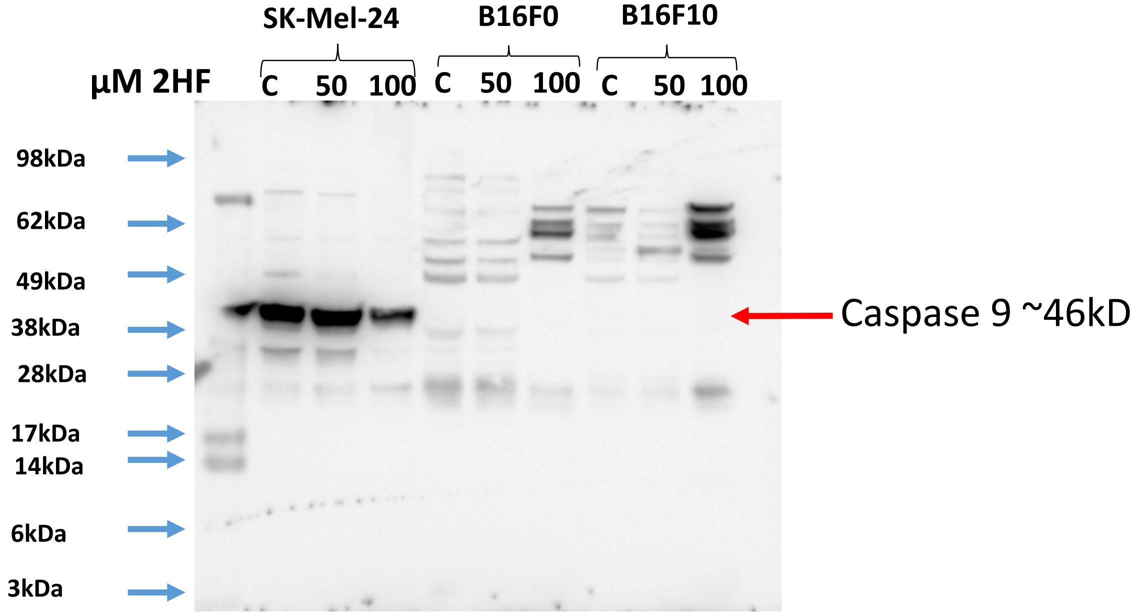

Antibody: Caspase-3 (31A1067):sc-56053

Caspase-3

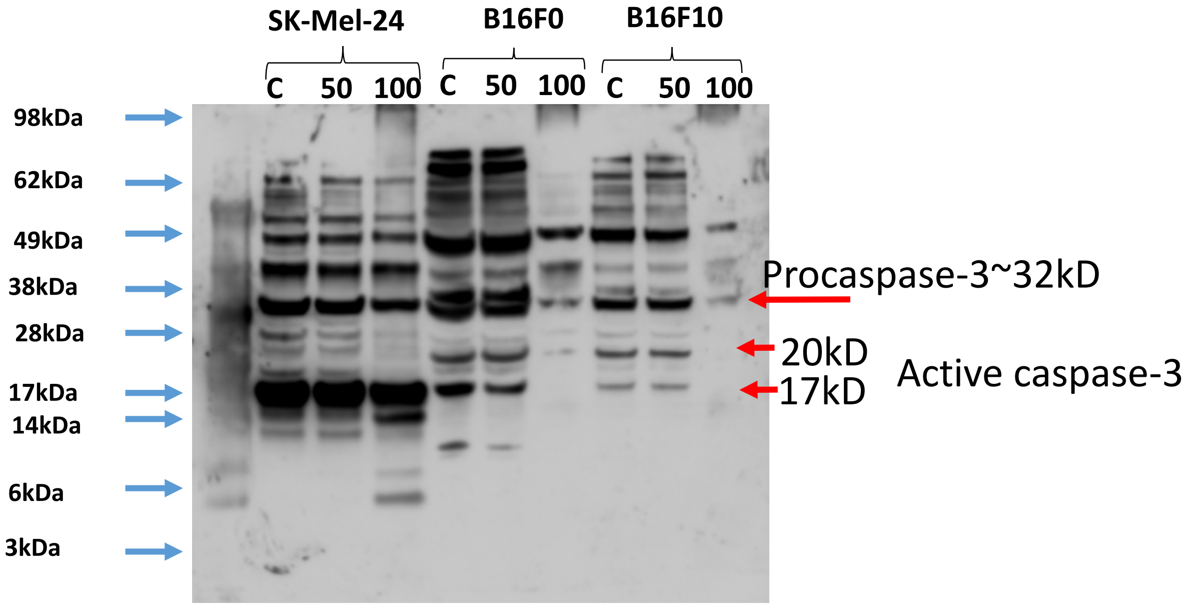

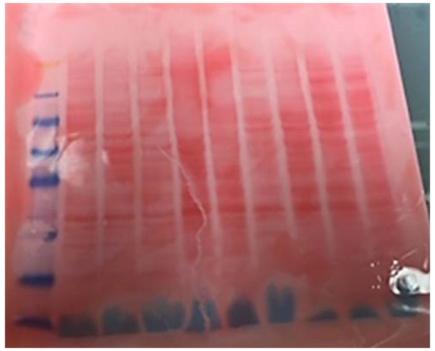

Ponceau stains for the gels to visualize the transferred proteins confirms equal protein loading

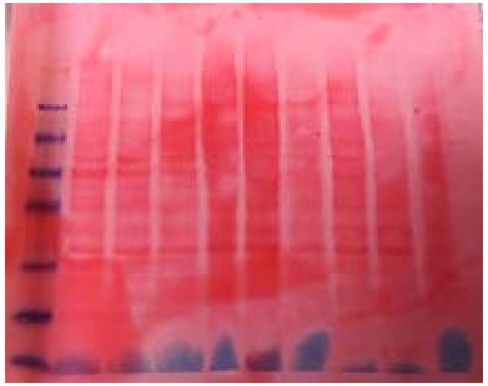

Supplemental Figure 6

Supplemental data for Figure 2C. Effect of 2HF on B16-F0, B16-F10 and SK-MEL-24 cells. Representative unprocessed western blots of control and 2HF treated animal tumors as shown in figure 9A. Red Arrow show cropped area included in figure 9A. Light blue arrows show molecular weight markers.

PARP-1

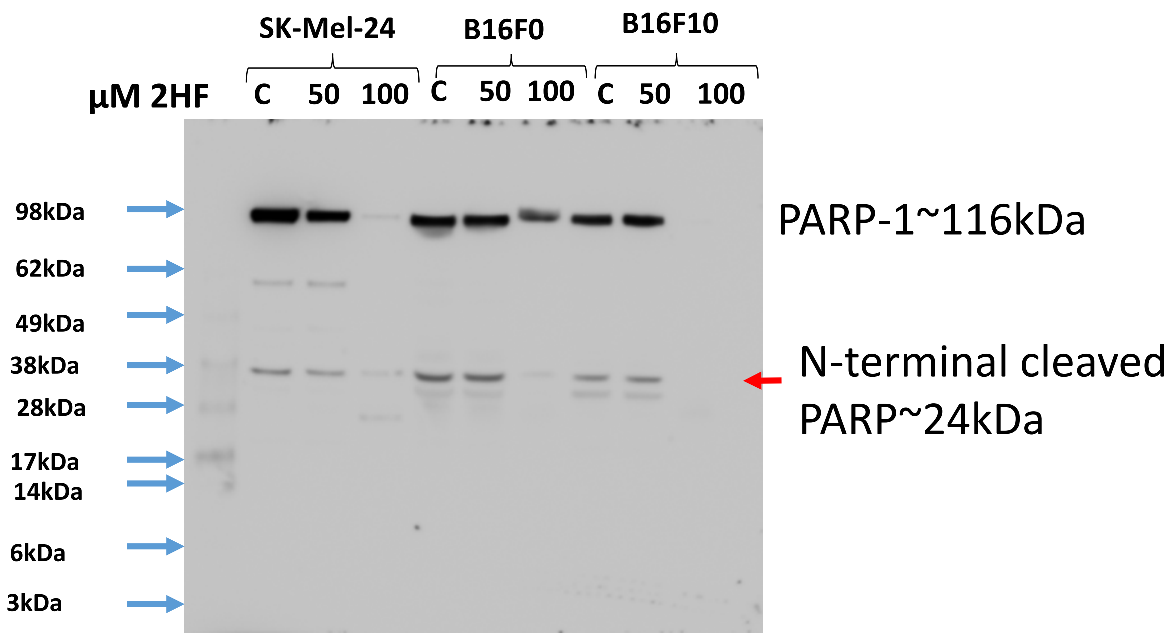

Antibody: PARP-1(B-10):SC-74470

Cleaved PARP-1

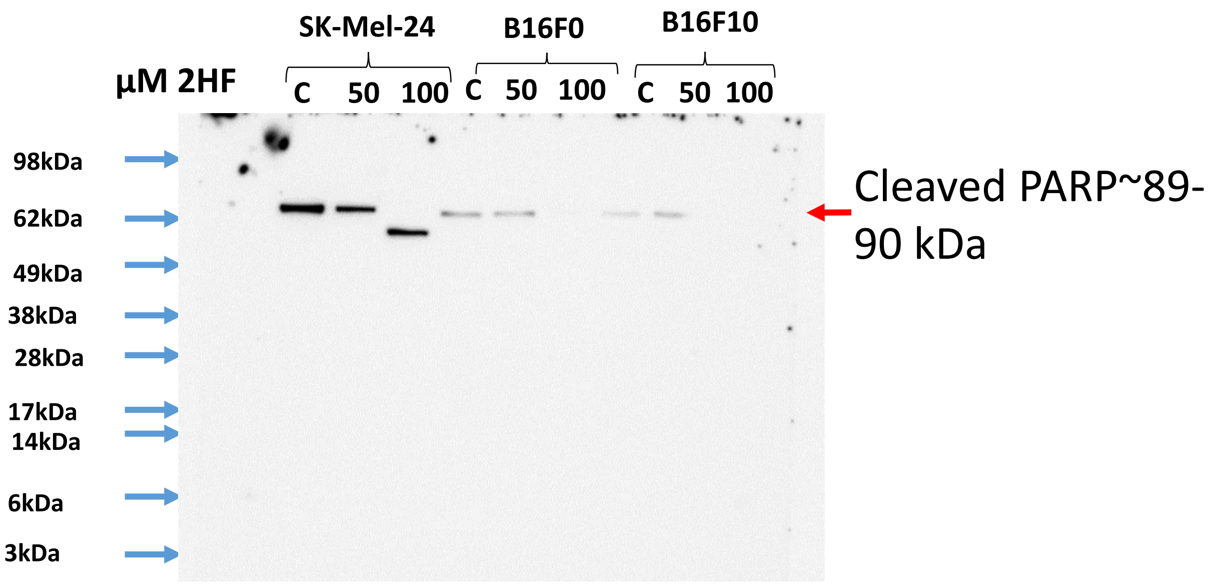

Antibody: Cleaved PARP-1 (194C1439)sc-56196

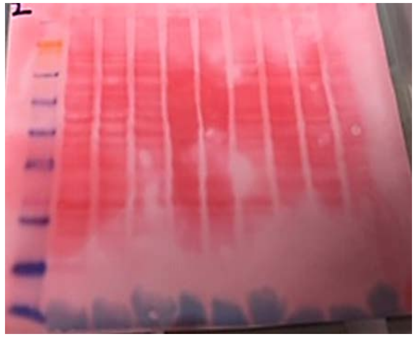

Ponceau stains for the gels to visualize the transferred proteins confirms equal protein loading

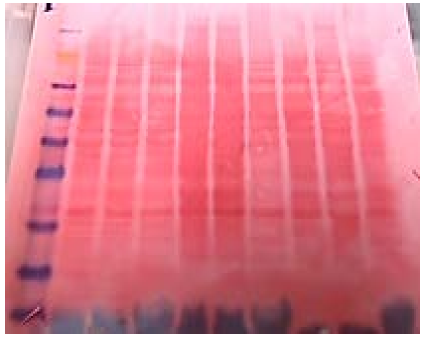

Supplemental Figure 7

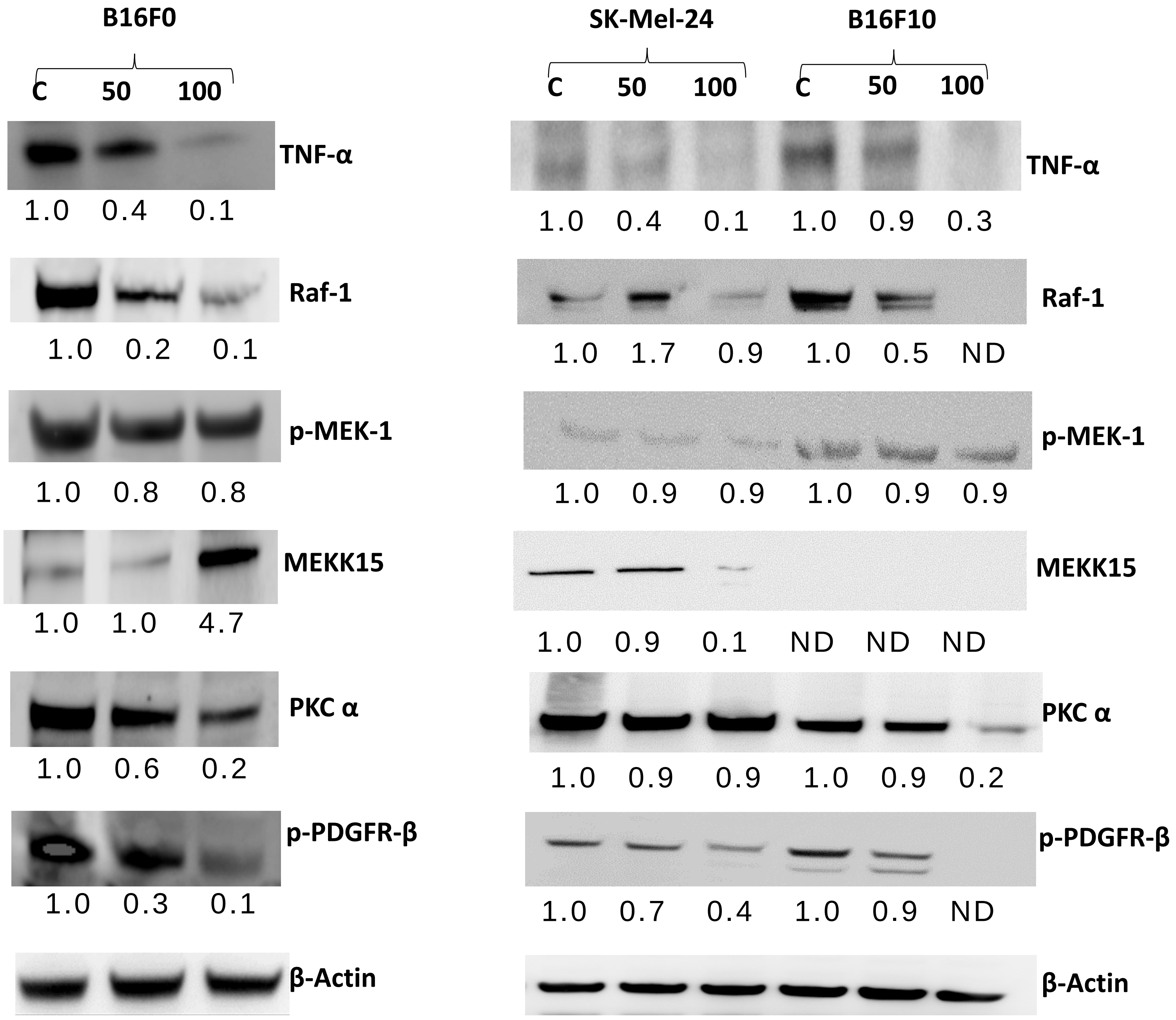

Supplemental data for Fig. 5.

Numbers below of each blot represent protein band intensity ratios to adequate controls after normalization against β-Actin.

Full blots are presented in next 4 figures.

Intracellular signaling markers in SK-Mel-24 and B16F10 cells after treatment with 50, 100 µM 2HF for 48h. Cells were plated in complete growth medium. After overnight, cells were treated with different doses of 2HF, and incubated 48h. Total cell lysates (25 µg/lane) were loaded on 4-12% bis-tris gel, with 1X MES gel running buffer. Proteins were transferred to nitrocellulose membrane, nonspecific binding was done in 1X clear milk, +0.1%T20 (Invitrogen) for 1h at room temperature. All primary antibodies were from (mouse monoclonal, except TNF-α (American Hamster), Santacruze Biotechnology (1:1000), in 1X clear milk +0.1%T20 overnight in 4^{0}C. Secondary antibody from Santacruze Biotech (1:2000) for 1h at room temperature in 1X clear milk, 0.1% T20.

Supplemental Figure 8

Supplemental data for Figure 5. Effect of 2HF on B16-F10 and SK-MEL-24 cells. Representative unprocessed western blots of control and 2HF treated animal tumors as shown in figure 9A. Red Arrow show cropped area included in figure 9A.

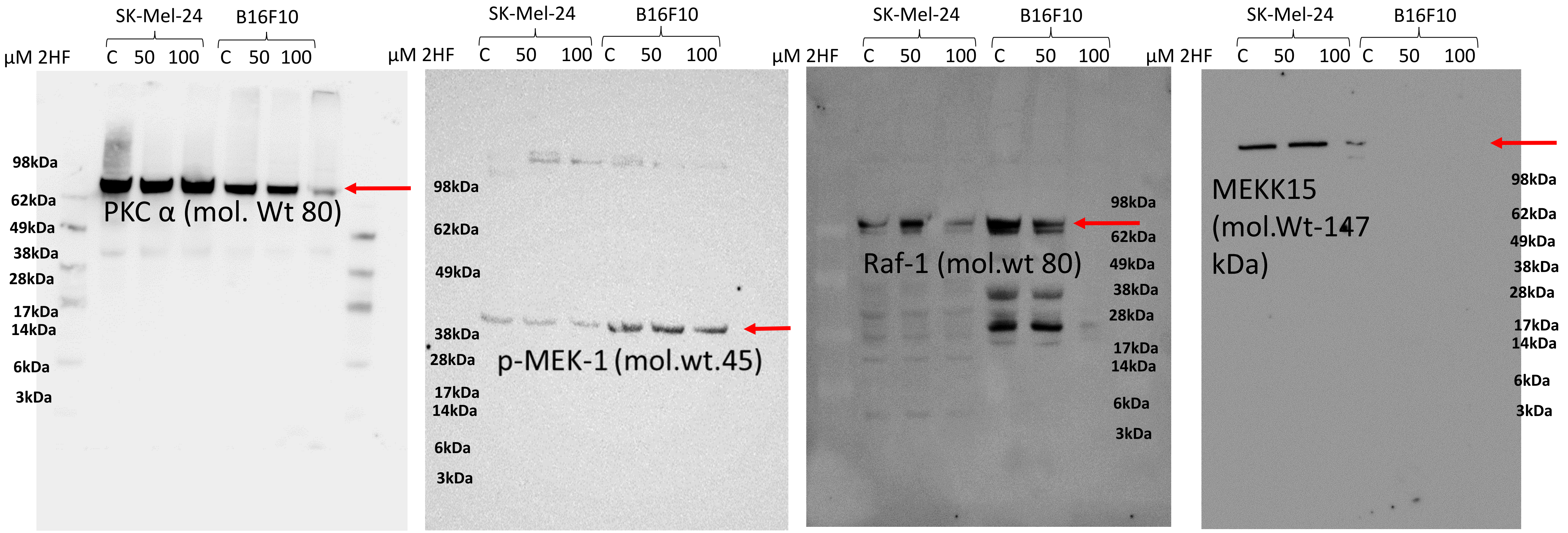

(PKC α Antibody (H-7): sc-8393)

(p-MEK-1 Antibody (47.Ser 222) : sc-136542)

Raf-1 Antibody (E-10): sc-7267

(MEKK 15 Antibody (D-6): sc-514606)

Supplemental Figure 9

Supplemental data for Figure 5. Effect of 2HF on B16-F10 and SK-MEL-24 cells. Representative unprocessed western blots of control and 2HF treated animal tumors as shown in figure 9A. Red Arrow show cropped area included in figure 9A.

PDGFR

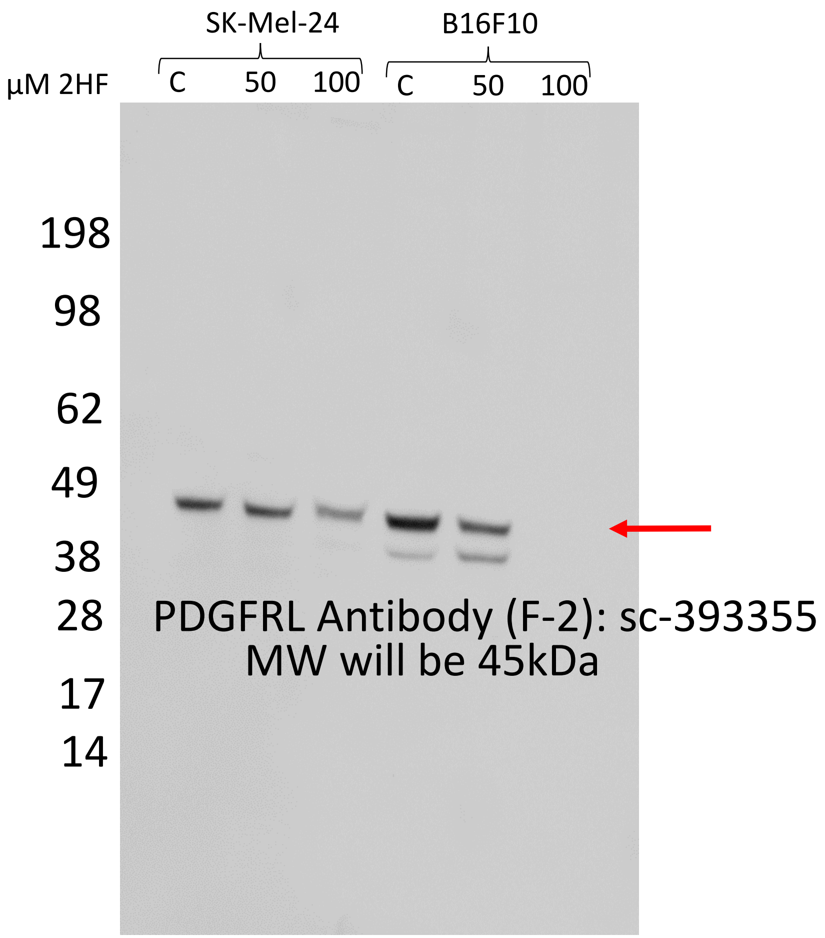

TNF-α

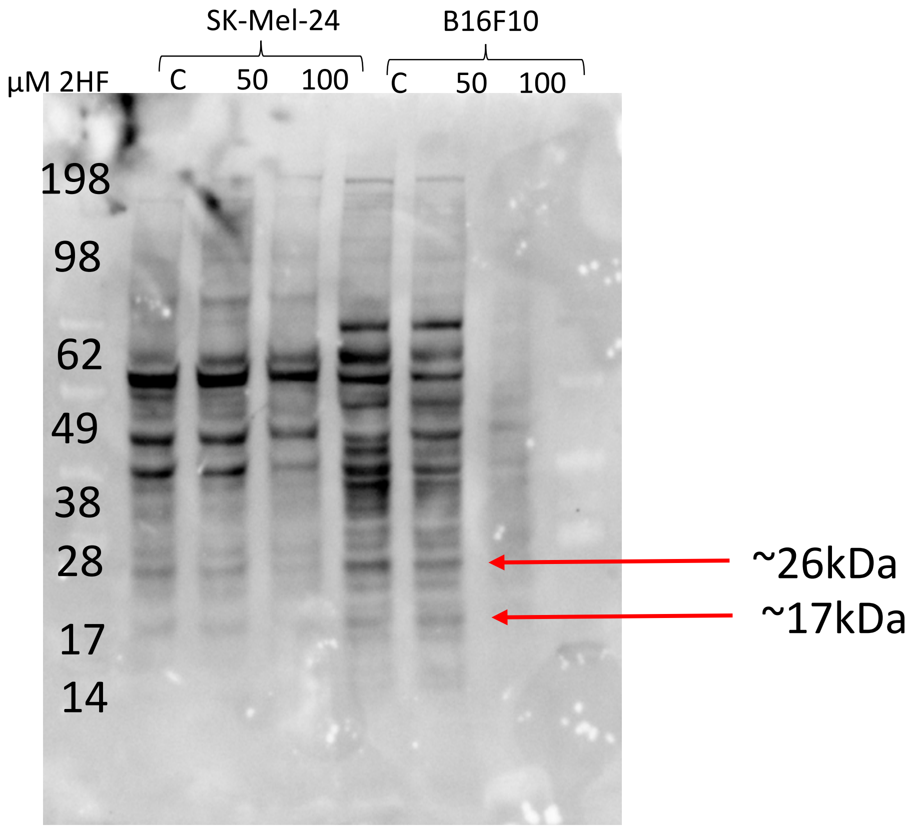

TNFα Antibody (TN3-19.12): sc-12744

Supplemental Figure 10

Supplemental data for Figure 5. Effect of 2HF on B16-F0

Representative unprocessed western blots of control and 2HF treated B16F0 cells as shown in figure 5. Red Arrow show cropped area included in figure 5. Light blue arrows show molecular weight markers.

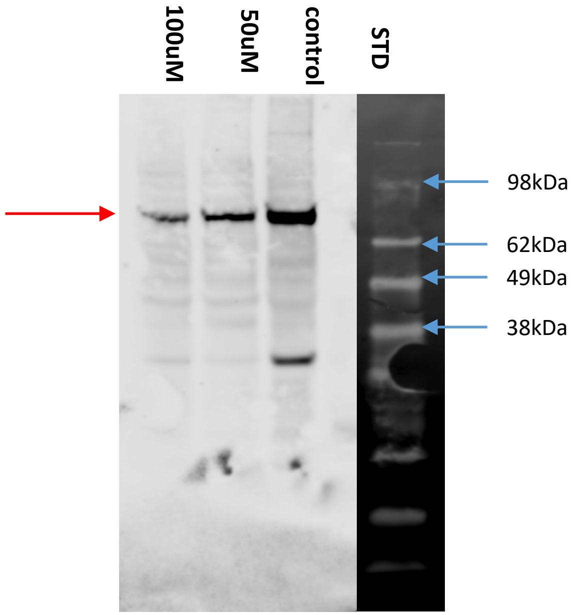

Raf-1(E-10)
M.W=80kDa

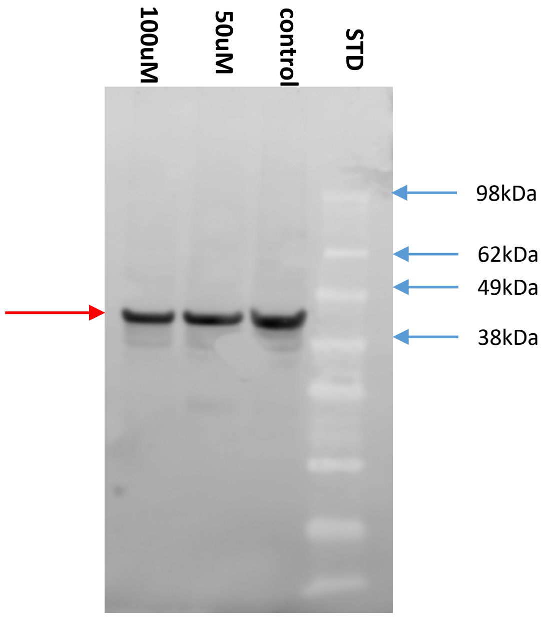

p-MEK-1(47.Ser222)
M.W=45kDa

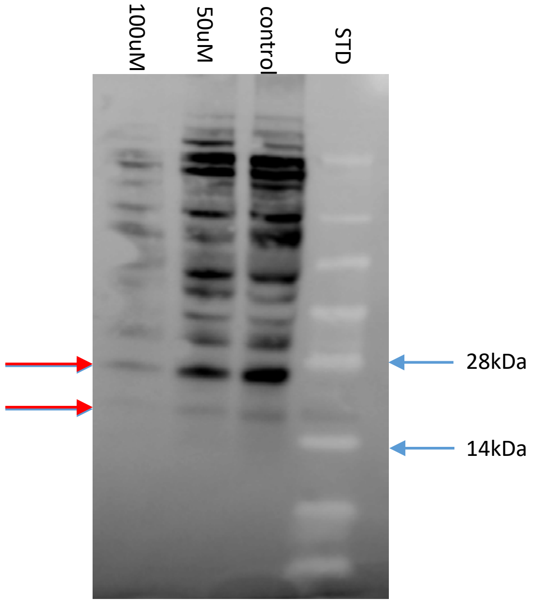

TNFα
M.W=26kDa(transmembrane TNFα)
M.W= 17kDa(soluble TNFα)

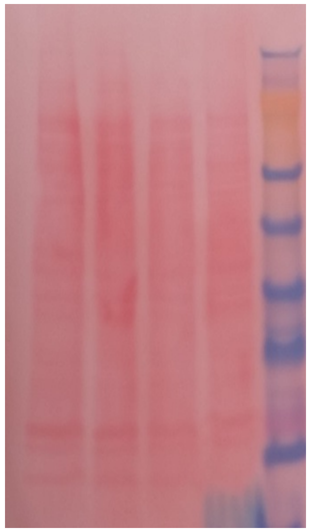

Ponceau stains for the gels to visualize the transferred proteins confirms equal protein loading

Supplemental Figure 11

Supplemental data for Figure 5. Effect of 2HF on B16-F0

Representative unprocessed western blots of control and 2HF treated B16F0 cells as shown in figure 5. Red Arrow show cropped area included in figure 5. Light blue arrows show molecular weight markers.

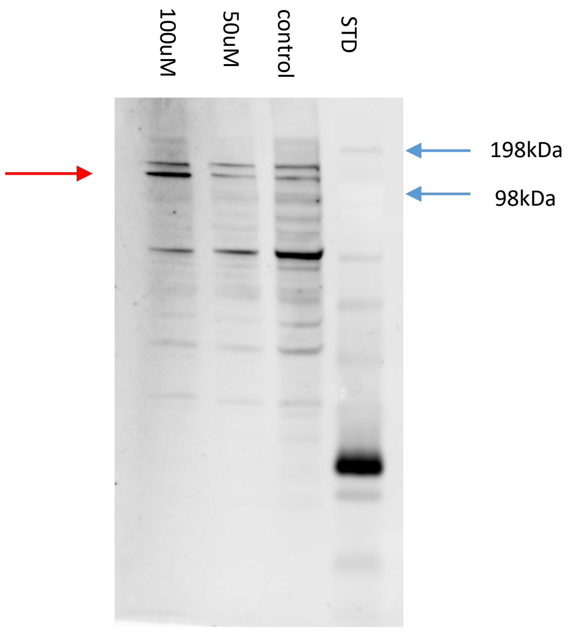

MEKK 15
M.W=147kDa

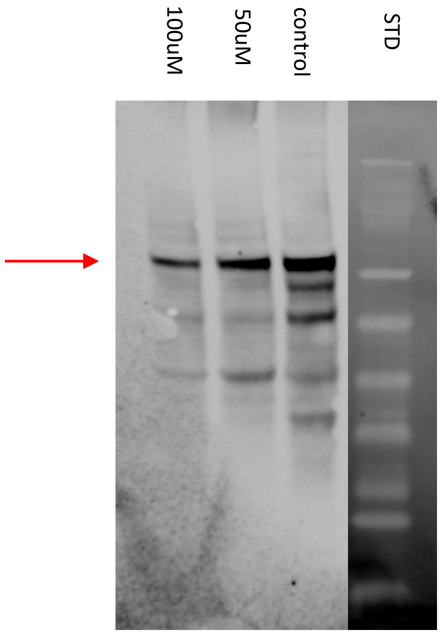

PKC(A-3)
M.W=80kDa

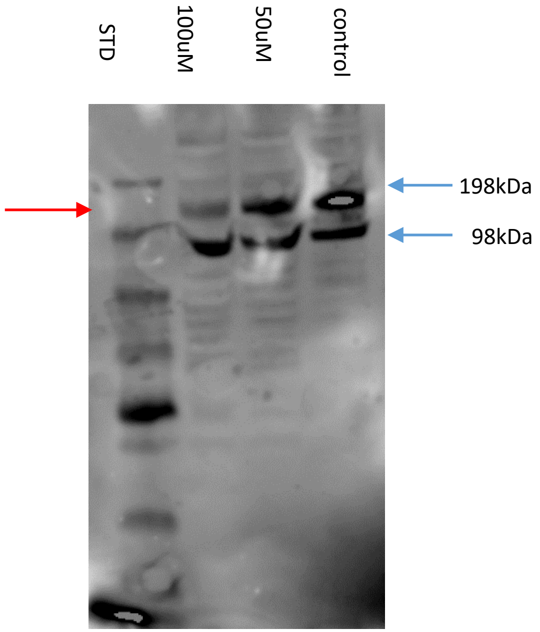

P-PDGFR-β

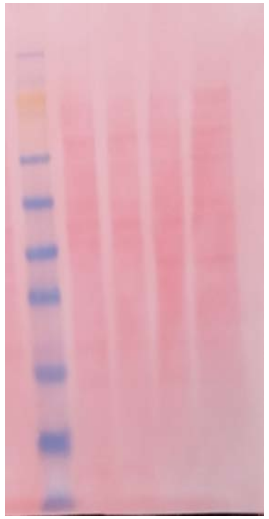

Ponceau stains for the gels to visualize the transferred proteins confirms equal protein loading

Supplemental Figure 12

Supplemental data for Figure 9A.

Numbers below of each blot represent protein band intensity ratios to adequate controls after normalization against GAPDH. **Full Blots are presented in next 2 figures.**

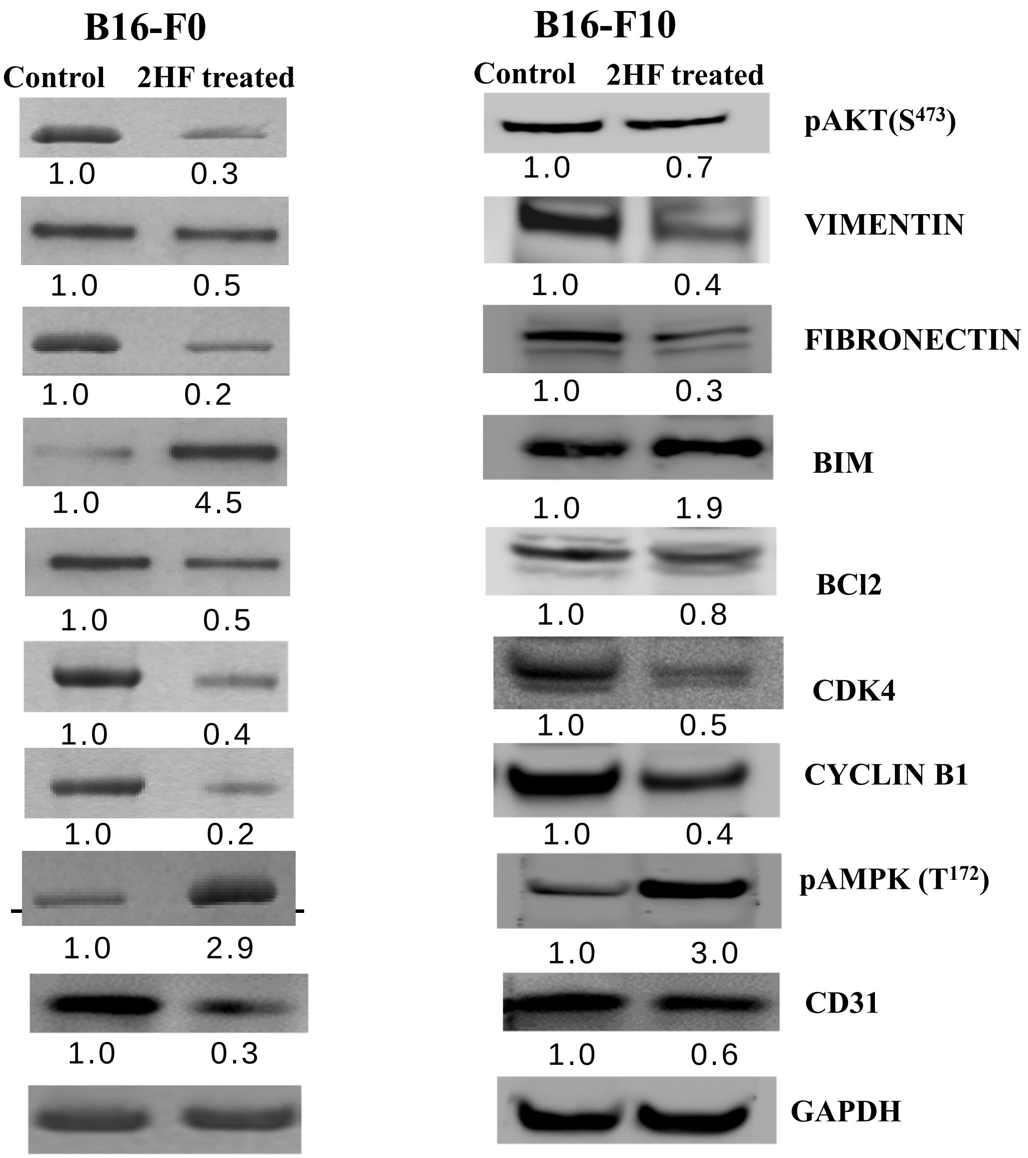

Supplemental Figure 13

Supplemental data for Figure 9A.

Representative unprocessed western blots of control and 2HF treated animal B16-F10 tumors as shown in figure 9A. Red Arrow show cropped area included in figure 9A. Light blue arrows show molecular weight markers.

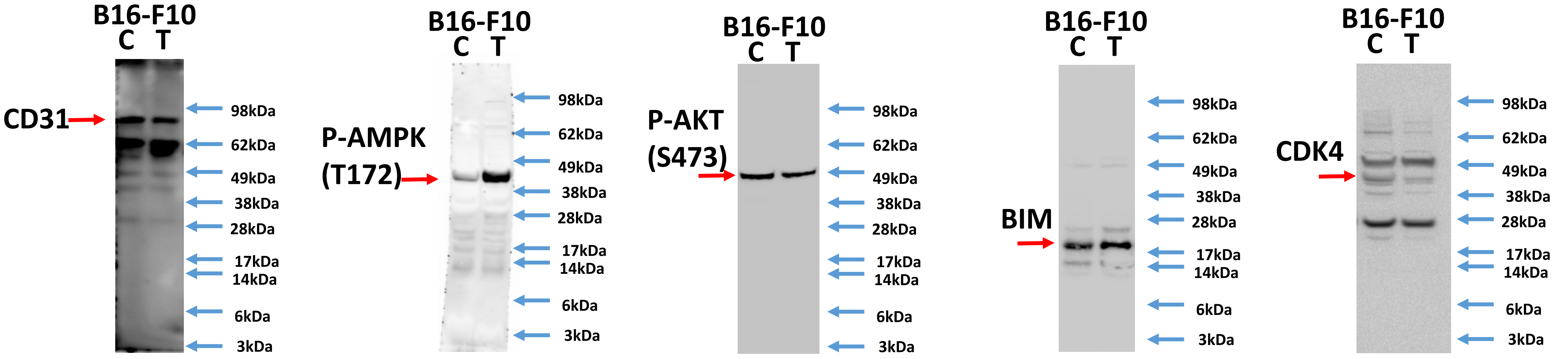

Supplemental Figure 14

Supplemental data for Figure 9A.

Representative unprocessed western blots of control and 2HF treated animal B16-F10 tumors as shown in figure 9A. Red Arrow shows cropped area included in figure 9A. Light blue arrows show molecular weight markers.

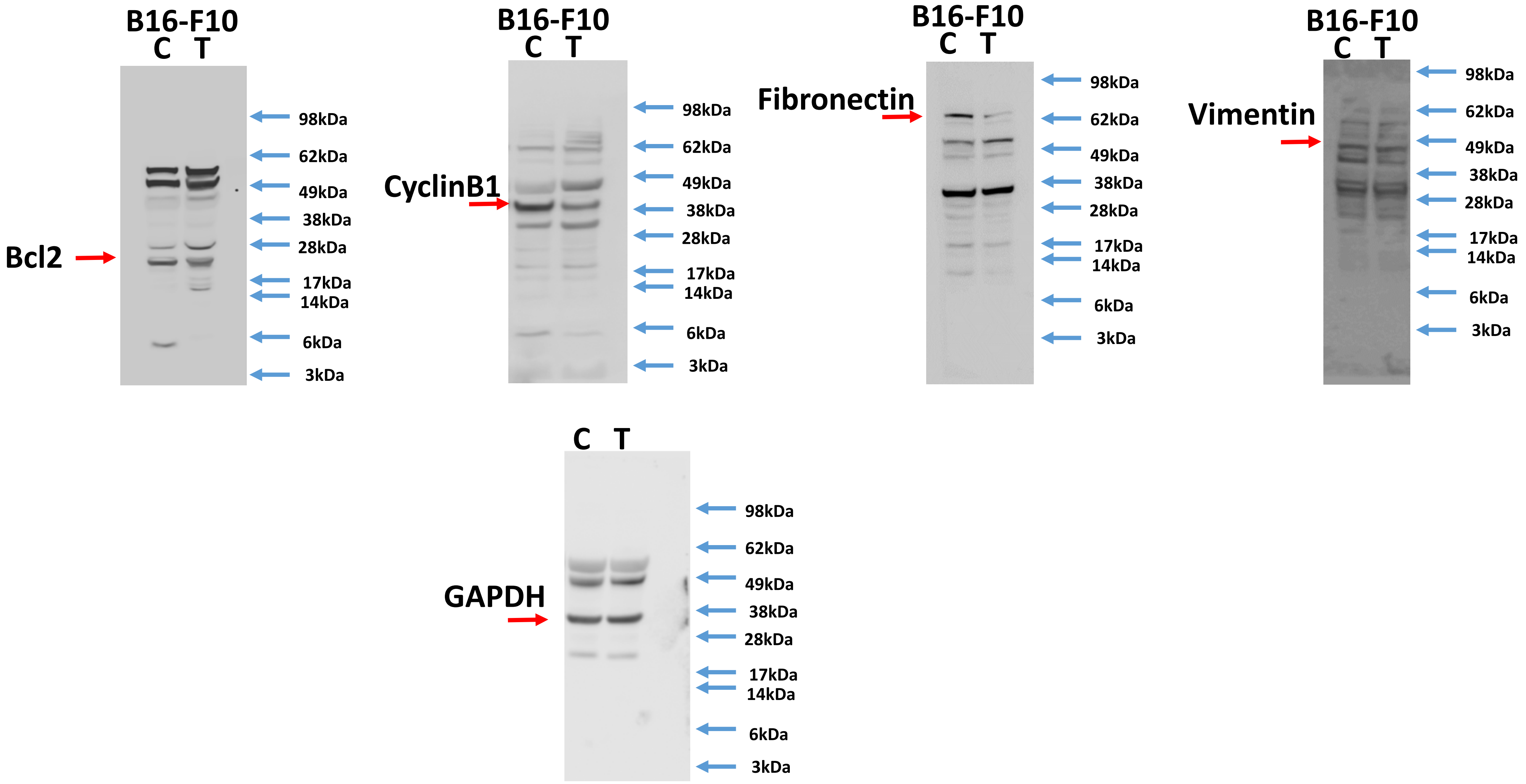

Supplement: Supplementary File 1 [file cancers-11-01556-s001.pdf]
